# FOXM1/lncRNA TYMSOS/miR-214-3p–Mediated High Expression of NCAPG Correlates With Poor Prognosis and Cell Proliferation in Non–Small Cell Lung Carcinoma

**DOI:** 10.3389/fmolb.2021.785767

**Published:** 2022-02-08

**Authors:** Yixiao Yuan, Xiulin Jiang, Lin Tang, Juan Wang, Dahang Zhang, William C. Cho, Lincan Duan

**Affiliations:** ^1^ The Department of Thoracic Surgery, The Third Affiliated Hospital of Kunming Medical University, Kunming, China; ^2^ Key Laboratory of Animal Models and Human Disease Mechanisms of Chinese Academy of Sciences/ Kunming Institute of Zoology, Kunming, China; ^3^ Department of Clinical Oncology, Queen Elizabeth Hospital, Hong Kong, Hong Kong SAR, China

**Keywords:** NSCLC, NCAPG, TYMSOS, miRNA-214-3p, tumor immune infiltration, cancer progression

## Abstract

Lung cancer is the most common cancer with high mortality. Increasing evidence has demonstrated that nonstructural maintenance of chromosomes condensin I complex subunit G (NCAPG) plays a crucial role in the progression of human cancers. However, the biological function and underlying mechanism of NCAPG in non–small cell lung cancer (NSCLC) are still unclear. Here, we utilized diverse public databases to analyze the expression of NCAPG in pan-cancer. We found that NCAPG was highly expressed in various human cancers, especially in NSCLC. NCAPG expression was significantly positively correlated with poor clinical-pathological features, poor prognosis, tumor mutational burden, DNA microsatellite instability, and immune cell infiltration in NSCLC. In addition, our results showed that depletion of NCAPG significantly inhibited NSCLC cell proliferation, migration, and self-renewal abilities, yet these could be reversed by adding microRNA (miRNA)-214-3p. Knockdown of long noncoding RNA (lncRNA) thymidylate synthetase opposite strand (TYMSOS) also inhibits the NSCLC cell proliferation, migration, and self-renewal abilities. In summary, our findings demonstrated that the crucial roles of the FOXM1/lncRNA-TYMSOS/miRNA-214-3p/NCAPG axis in NSCLC may shed light on how NCAPG may act as a therapeutic target for NSCLC.

## Introduction

Lung cancer is the major cause of cancer-related death worldwide, according to cancer statistics in 2020, the morbidity rate of lung cancer ranks second, whereas the death rate of lung cancer ranks the top (Siegel et al., 2020). Lung cancer is composed of non–small cell lung carcinoma (NSCLC) and small cell lung carcinoma, NSCLC includes lung adenocarcinoma (LUAD), lung squamous cell carcinoma, and large-cell lung carcinoma, the NSCLC accounts for approximately 85% of all cases (Molina et al., 2008; Fan et al., 2021). The high lung cancer mortality is owing to cancer metastatic with the time of diagnosis, indicating that ameliorating patient’s survival will require more effective systemic therapies (Morgensztern et al., 2010). However, there is much progress achieved in cancer treatment methods, ranging from targeted therapies to immunotherapy (Herbst et al., 2018); the survival period of lung cancer patients was very short (Xu et al., 2020a; Liang et al., 2021). Therefore, finding a new therapeutic target or biomarker is essential for effectively preventing the development of lung cancer.

Recently, studies have started to characterize the regulatory effects that condensin complexes may have on diverse cancers, including colon adenocarcinoma and liver hepatocellular carcinoma. These condensin complexes play a different role in the progression of cancer, as well as drug resistance (Wu et al., 2019). Studies have indicated that nonstructural maintenance of chromosomes condensin I complex subunit G (NCAPG) was essential for chromatin separation (Eberlein et al., 2009). Its elevated expression has been reported in several cancers, including hepatocellular carcinoma (HCC) (Gong et al., 2019), gastric cancer (Sun et al., 2020), ovarian cancer (Xu et al., 2020b), and cardia adenocarcinoma (Zhang et al., 2021). It has been shown that elevated NCAPG level promotes cancer cell migration by activating Wnt/β-catenin signaling (Zhang et al., 2021). A recent study found that NCAPG through activating the PI3K/AKT pathway promotes HCC cell proliferation and antiapoptosis (Gong et al., 2019). Furthermore, up-regulation of NCAPG expression significantly facilitated the progression of BRCA. It has been shown that NCAPG regulated the stability of STAT3 confers trastuzumab resistance in HER2-positive breast cancer (Jiang et al., 2020). In gastric cancer, Song et al. reported that miR-193b-3p via targets the NCAPG and reduces the expression of NCAPG, resulting in inhibition of the cell proliferation of gastric cancer (Song et al., 2018). Moreover, up-regulated NCAPG expression is also linked to the cancer pathological stage of castration-resistant prostate cancer; knockdown of NCAPG inhibited cancer cell aggressiveness (Arai et al., 2018). However, a comprehensive analysis of the function and regulation mechanism of NCAPG in LUAD is still insufficient. Thus, our study utilized various bioinformatics approaches and functional studies to examine whether NCAPG is involved in NSCLC progression, immune infiltration, and its plausible molecular regulation.

In this study, we compared the expression of NCAPG between NSCLC tissues and normal samples and investigated the correlation between NCAPG expression and clinical parameters of NSCLC. In addition, we explored the prognostic value and clinical significance of NCAPG in NSCLC. Meanwhile, the correlation between NCAPG expression and immune infiltration was analyzed to explore the potential mechanisms involved in NCAPG modulation in the progression of NSCLC. Finally, the biological role of NCAPG was identified in NSCLC. In summary, we demonstrated the potential role of NCAPG in regulating tumor progression and its potential application in the diagnosis and prognostic evaluation in NSCLC.

## Materials and Methods

### Data Collection

The Cancer Genome Atlas (TCGA)–LUAD cohort data and corresponding clinical information of 535 LUAD patients were downloaded from the TCGA website (https://portal.gdc.cancer.gov/repository). LUAD patients were classified into low- and high-NCAPG expression groups according to the median NCAPG expression value. NCAPG expression data and clinical information from datasets GSE10072 and GSE18842 were downloaded from the GEO database and validated for survival analyses. The gene expression profiles were normalized using the scale method provided in the “limma” R package. Data analysis was performed with the R (version 3.6.3) and ggplot2 (3.3.3) packages. The expression data were normalized to transcripts per kilobase million values before further analysis. Besides, the ROC curve was used to evaluate the diagnostic value of NCAPG L using the pROC R package and ggplot2 R package.

### Construction and Evaluation of the Nomogram

The RMS R package was performed to generate a nomogram. C-index and calibration curve were performed by the Hmisc R package. In our study, C-index was performed to determine the discrimination of nomogram and used a bootstrap method with 1,000 resamples to calculate C-index.

### Gene Expression Profiling Interactive Analysis

Gene Expression Profiling Interactive Analysis (GEPIA) (http://gepia.cancer-pku.cn/index.html) is a user-friendly web portal for gene expression analysis based on TCGA and GTEx data (Tang et al., 2017). In the current study, GEPIA was used to analyze the expression and prognosis values of NCAPG in pan-cancer. Furthermore, we used GEPIA analysis of the correlation between NCAPG expression and pathological stage.

### Kaplan–Meier Plotter Database Analysis

We used KM Plotter (http://kmplot.com), an online database that contains gene expression data and survival information of 3,452 clinical lung cancer patients, to analyze the prognostic value of NCAPG in lung cancer (Győrffy, 2021). The patient samples were separated into two groups by median expression (high expression and low expression) to analyze the overall survival (OS) with hazard ratios (HRs) with 95% confidence intervals and log-rank *p* values.

### Immune Infiltration Analysis

TIMER (https://cistrome.shinyapps.io/timer/) web server is a comprehensive resource for systematical analysis of immune infiltrates across diverse cancer types. In this study, TIMER was used to examine the correlation between somatic copy number alterations of NCAPG and the infiltration level of B cells, CD4^+^ T cells, CD8^+^ T cells, neutrophils, macrophages, and dendritic cells.

### Function and Pathway Analysis by Gene Set Enrichment Analysis

In the present research, we utilized the linkedomics database (http://www.linkedomics.org/login.php) to obtain the coexpression genes of NCAPG in LGG. The gene set KEGG v6.2. symbols.gmt,” which served as a reference gene set, was downloaded from the Molecular Signatures Database (MSigDB) (http://software.broadinstitute.org/gsea/msigdb). We used the Gene Set Enrichment Analysis (GSEA) software and clusterProfiler package to perform the Gene Ontology (GO) and Kyoto Encyclopedia of Genes and Genomes (KEGG) enrichment analysis signaling pathway of NCAPG in lung cancer (Subramanian et al., 2005; Yu et al., 2012; Vasaikar et al., 2018).

### The NCAPG-Interacting Genes and Protein Analysis

The GeneMANIA database (http://www.genemania.org) was applied to construct the NCAPG interaction network (Zuberi et al., 2013). The STRING online database (https://string-db.org/) was used to construct a protein–protein interaction (PPI) network of NCAPG (Szklarczyk et al., 2019).

### Prediction of Long Noncoding RNA and c8eRNA Network Construction

We used the starBase (http://starbase.sysu.edu.cn/) to predict the potential upstream microRNAs (miRNAs) of NCAPG and examine the expression, prognosis, and correlation between the miRNA-214-3p and long noncoding RNA (lncRNA); we used the starBase to predict the binding sites among the miRNA, mRNA, and lncRNA (Li et al., 2014). The lncLocator (www.csbio.sjtu.edu.cn/bioinf/lncLocator.), a subcellular localization predictor for lncRNAs based on a stacked ensemble classifier, and CPC2 (http://cpc2.cbi.pku.edu.cn), a fast and accurate coding potential calculator based on sequence intrinsic features (Kang et al., 2017; Cao et al., 2018). In this study, lncLocator and CPC2 were used to explore the subcellular localization and the protein-coding ability of thymidylate synthetase opposite strand (TYMSOS).

### Cell Culture, RNA Isolation, and Real-Time Polymerase Chain Reaction

Cell culture, RNA isolation, and real-time polymerase chain reaction (PCR) assay were performed as published (Shi et al., 2019). The BEAS-2B cell line was purchased from the cell bank of Kunming Institute of Zoology and cultured in BEGM media (Lonza, CC-3170). HEK-293T was obtained from ATCC. Lung cancer cell lines, including A549, H1299, and H1975, were purchased from Cobioer (China) with STR documents; A549, H1299, and H1975 cells were all cultured in RPMI 1640 medium (Corning) supplemented with 10% fetal bovine serum (FBS) and 1% penicillin/streptomycin. HEK-293T cells were cultured in a Dulbecco modified eagle medium (Corning). The detail information of primer used in this study are as follows: NCAPG-F: GAG​GCT​GCT​GTC​GAT​TAA​GGA, NCAPG-R: AAC​TGT​CTT​ATC​ATC​CAT​CGT​GC, TYMSOS-F: ATG​ACG​CCC​GCC​TCG​GGG​GCC, TYMSOS-R: TCA​GGA​AGG​ACG​ACC​GCA​CGG​GCA​CC, FOXM1-F: CGT​CGG​CCA​CTG​ATT​CTC​AAA, FOXM1-R: GGC​AGG​GGA​TCT​CTT​AGG​TTC, miRNA-214-3p-F TTT​TTA​CTA​CTA​TGG​CGG​GTG​ATA​AAA​CGT​GTA, miRNA-214-3p-R: GCA​AGC​TGT​AAT​CGA​CGG​GAA​GAG​CAT​GCC​CAT​CC, ACTIN-F: CTTCGCGGGCGACGAT, and ACTIN-R: CCA​TAG​GAA​TCC​TTC​TGA​CC. The expression quantification was obtained with the 2^−ΔΔCt^ method.

### Constructs, Transfection, and Infection

The FOXM1 and NCAPG shRNAs and control scrambled shRNA were transfected into HEK-293 T cells with the psPAX2/pMD2 G plasmids (Addgene) using calcium phosphate. After transfection, the cell supernatants were harvested and used to infect the A549 cell, and the stably lentiviral infected cells were selected with puromycin. Two shRNAs for knockdown of TYMSOS are as follows (sh-TYMSOS#1: 5ʹ-TAAGAAGATCTAATGCATCCT-3ʹand sh-TYMSOS#2: 5ʹ-AAC​ACT​TTA​TTA​TCA​CAT​CAG-3ʹ) were used to silence TYMSOS. Two shRNAs for knockdown of FOXM1 are as follows: sh-FOXM1 #1: 5ʹ-TCC​TGG​AGG​CTC​ACG​CCC​CCA-3ʹ and sh-FOXM1#2:5ʹ-GCCACAGGTTTCTGGCCTTGC-3ʹ, used to silence FOXM1.

### Cell Proliferation, Colony Formation, and Tumorsphere Formation Assays

Cell proliferation assay was performed as previously described (Xu et al., 2020a). Indicated tumor cells were plated onto 12-well plates; the cell numbers were subsequently counted each day using an automatic cell analyzer countstar (Shanghai Ruiyu Biotech Co.). For colony formation assay, a total of 500 indicated cells/well were plated onto 6-well plates and cultured for 2 weeks at 37°C. The medium was changed every 3 days. Two weeks later, indicated cells were fixed with 4% paraformaldehyde for 30 min at room temperature and subsequently stained with 0.1% crystal violet for 30 min at room temperature.

### Cell Migration Assay

The cell migration assay was performed as documented (Shi et al., 2019). For wound healing assay, to produce a wound, the monolayer cells in 6-well plate were scraped in a straight line with pipette tips. Plate was then washed with warm PBS to remove detached cells. Photographs of the scratch were taken at indicated time points using Nikon inverted microscope. For Transwell assay, 2.5 × 10^4^ cells in 100 µL serum-free medium were plated in 24-well plate chamber insert (Corning Life Sciences, catalog no. 3422), with medium containing 10% FBS at the bottom of the insert. Cells were incubated for 24 h and then fixed with 4% paraformaldehyde for 20 min. After washing, cells were stained with 0.5% crystal violet blue. The positively stained cells were examined under the light microscope.

### Nuclear and Cytoplasmic Extraction

The nuclear and cytoplasmic fractions of RNA were extracted with a PARIS™ kit (Invitrogen, Thermo Fisher Scientific, Waltham, MA, USA). The cell slide was placed at the bottom of the 6-well plate, and an appropriate number of cells (3 × 10^5^/well) were cultivated. Before the experiment, the cell confluence reached 60%–70%.

### Luciferase Activity Assays

The sequences of TYMSOS, NCAPG–3′ UTR, and their miR-214-3p binding sites mutant versions were synthesized and added to luciferase reporter vector pGL3 (Promega, Shanghai, China), named TYMSOS-WT, TYMSOS-Mut, NCAPG-WT, and NCAPG-Mut, respectively. The experimental steps follow the manufacturer’s protocols of Dual Luciferase Assay Kit (Promega).

### Immunohistochemistry Staining

Immunohistochemistry (IHC) staining was performed as documented (Kang et al., 2017). Briefly, cancer tissues were collected; primary antibody overnight incubation and second antibody incubation were performed. Finally, develop using the instrument. The detailed information of primers used in this study is as follows: (NCAPG, catalog no. 24563-1-AP, 1:200; Proteinch, Shanghai, China). Lung cancer tissue and normal lung tissues were obtained from advanced-stage lung cancer patients from the Third Affiliated Hospital of Kunming Medical University (Yunnan Tumor Hospital), as well as ethics approval and consent to participate. This study was approved by the ethics committee of the Third Affiliated Hospital of Kunming Medical University (Yunnan Tumor Hospital), and informed consent was obtained from all patients.

### Statistical Analysis

The results of Kaplan–Meier plots, PrognoScan, and GEPIA are displayed with HR and P or Cox *p* values from a log-rank test. The Wilcoxon signed-rank test and Wilcoxon rank-sum test were performed to investigate the expression of NCAPG in paired and nonpaired samples, respectively. The Wilcoxon signed-rank test was used to analyze the relations between NCAPG expression and clinical features. The univariate and multivariate analyses using the Cox regression model were carried out to evaluate death risk, including gender, age, number of pack-years smoked, T stage, N stage, M stage, pathological stage, and NCAPG expression. All the tests were two-sided, and *p* < 0.05 was considered statistically significant. *p* < 0.05 (*), *p* < 0.01 (**), and *p* < 0.001 (***) were considered significant.

## Results

### Expression Level, Clinical-Pathological Features, and Prognosis of NCAPG in Pan-Cancer

**FIGURE 1:**
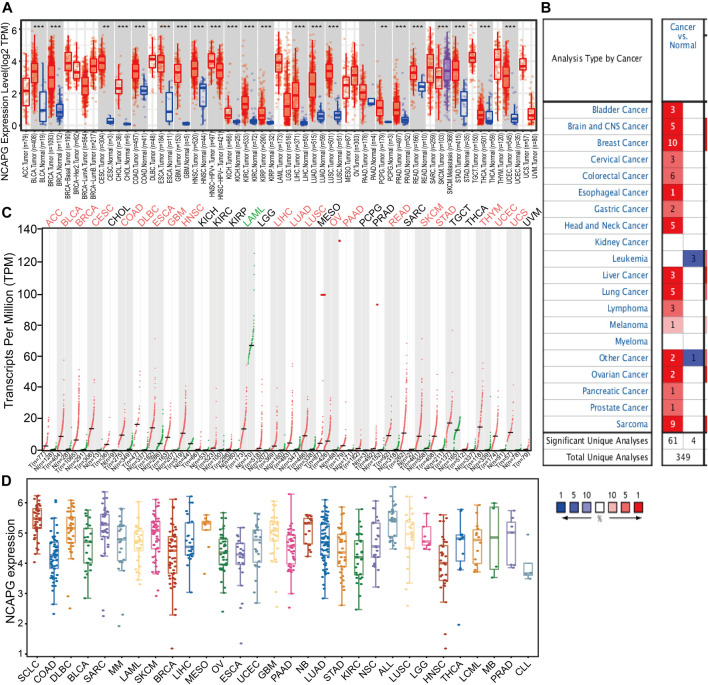
Expression analysis for NCAPG in human cancers **(A)** NCAPG expression of different tumor types in pan-cancer examined by the TIMER database. **(B)** Increased expression of NCAPG in pan-cancer examined by the Oncomine database. **(C)** NCAPG expression in pan-cancer examined by TCGA and GTEx data. **(D)** The expression of NCAPG in different tumor cells lines was examined by the CCLE database. *p < 0.05, **p < 0.01, ***p < 0.001.

To examine the expression of NCAPG in various cancers, we applies the TIMER database to analyze the expression of NCAPG in human cancer. The results revealed that NCAPG was significantly up-regulated in 21 cancer types than normal samples, including bladder urothelial carcinoma, breast invasive carcinoma, cervical squamous cell carcinoma and endocervical adenocarcinoma, cholangiocarcinoma, colon adenocarcinoma, esophageal carcinoma, glioblastoma multiforme, head and neck squamous cell carcinoma, kidney chromophobe, kidney renal clear cell carcinoma, kidney renal papillary cell carcinoma, liver hepatocellular carcinoma, LUAD, lung squamous cell carcinoma, pheochromocytoma and paraganglioma, prostate adenocarcinoma, rectum adenocarcinoma, skin cutaneous melanoma, stomach adenocarcinoma, thyroid carcinoma, and uterine corpus endometrial carcinoma were prominently ascended when matched to normal tissues (Figure 1A). However, no significant difference of NCAPG in lymphoid neoplasm diffuse large B-cell lymphoma, brain lower-grade glioma, ovarian serous cystadenocarcinoma, sarcoma, uterine carcinosarcoma, and uveal melanoma was observed. Next, we utilized the Oncomine database to examine the differences in expression of NCAPG in various cancers. The analysis result has shown that the expression of NCAPG elevated in 17 tumors compared with the normal tissues (Figure 1B). Besides, we showed that the expression of NCAPG was decreased in acute myeloid leukemia (LAML). Furthermore, we check the level of NCAPG in TCGA pan-cancers using the GEPIA tool. The result showed that NCAPG expression in ACC, BLCA, BRCA, CESC, COAD, DLBC, esophageal carcinoma, glioblastoma multiforme, HNSC, LIHC, LUAD, LUSC, OV, pancreatic adenocarcinoma, rectum adenocarcinoma, SKCM, STAD, THYM, UCEC, and UCS was significantly elevated in cancer than corresponding normal controls. And in LAML, NCAPG was decreased (Figure 1C). Finally, we used the CCLE database to analyze the expression of NCAPG in various tumor cells; the results indicated that NCAPG was elevated in LUAD cells (Figure 1D). Taken together, these results confirm that NCAPG plays a crucial role in carcinogenesis of the human cancer.

Given that NCAPG was highly expressed in diverse cancers, therefore, we next examined the expression of NCAPG whether correlated with the clinical-pathological features in NSCLC; we applied the GEPIA approach to analyze NCAPG expression and the pathological stages across various cancer types of TCGA. The results showed that high expression of NCAPG was significantly correlated with the pathological stages of human cancers, including ACC, BRCA, KIRC, KIRP, LIHC, LUAD, LUSC, OV, SKCM, TGCT, and THCA (Supplementary Figure S1).

Finally, survival time analysis of NCAPG in pan-cancer was conducted. Four prognostic indices, consisting of OS, disease-free survival (DFS), progression-free survival (PFS), and disease-specific survival (DSS) were contained. Regarding OS, up-regulation of NCAPG expression correlated with unfavorable prognosis in ACC, KIRC, KIRP, LGG, LIHC, LUAD, MESO, PAAD, PCPG, and SARC, but THYM patients elevated NCAPG expression exhibit good prognosis (Supplementary Figure S2). For DFS, about these varies cancer, up-regulation of NCAPG shown poor prognosis in ACC, KICH, KIRC, KIRP, LGG, LIHC, PAAD, PCPG, PRAD, SARC, THCA, and UVM (Supplementary Figure S3). Concerning PFS, in human cancers, up-regulation of NCAPG expression shows a bad prognosis in ACC, KICH, KIRC, KIRP, LGG, LIHC, MESO, PAAD, PCPG, PRAD, and UVM (Supplementary Figure S4A). Concerning DSS, elevated expression of NCAPG implies poor prognosis in CESC, KIRP, LIHC, MESO, SARC, and THCA (Supplementary Figure S4B). Through analysis of the OS, RFS, PFS, and DSS of NCAPG in human cancer, NCAPG may be used for an unfavorable prognostic biomarker in patients with ACC, KIRC, KIRP, LGG, LIHC, LUAD, MESO, PAAD, PCPG, and SARC. The above results demonstrated that NCAPG plays an important role in diverse cancer progression.

### Correlation Between NCAPG Expression and Tumor Mutational Burden, DNA Microsatellite Instability

Studies have demonstrated that tumor mutational burden (TMB), which represents the number of somatic mutations that occur on the coding region of the tumor cell genome, may be a biomarker of sensitivity to immune checkpoint inhibitors (Campbell et al., 2017; Chalmers et al., 2017; Hu et al., 2021). Microsatellite instability (MSI) refers to the random changes in microsatellites in tumor cells. These MSI changes have been linked to increased sensitivity to checkpoint inhibitor drugs (Cortes-Ciriano et al., 2017). We figured out the TMB and MSI of every sample in human cancer and then examined the relevance between NCAPG expression and TMB, and MSI in various human cancers. The analysis result indicated that the elevated expression of NCAPG correlated positively with TMB in ACC, UCEC, TGCT, STAD, SKCM, SARC, READ, PRAD, PAAD, OV, MESO, LUSC, LUAD, LGG, KIRC, KICH, HNSC, COAD, BRCA, and BLCA but notable negative within THYM (Supplementary Figure S5A). Furthermore, up-regulated expressions of NCAPG positively correlated with MSI in COAD, ESCA, LIHC, LUSC, SARC, STAD, UCEC, and UCS but negative in DLBC, LAML, and READ (Supplementary Figure S5B).

### The Expression of NCAPG in Immune and Molecular Subtypes of Human Cancers

Next, we use the TISIDB website to examine the expression of NCAPG in different immune and molecular subtypes in various tumors (Qiu et al., 2021). Immune subtype was including wound healing, IFN-gamma dominant, inflammatory, lymphocyte depleted, immunologically quit, TGF-b dominant. The results indicated that NCAPG expression was related to diverse immune subtypes in BLCA, BRCA, CESC, KIRC, KIRP, LIHC, LUAD, LUSC, MESO, OV, PRAD, SARC, SKCM, STAD, UVM, and UCEC (Supplementary Figure S6). For example, in LUAD, NCAPG had high expression in the wound healing group, IFN-gamma dominant group, and low expression in lymphocyte depleted types. For molecular subtypes of cancers, a positive connection with NCAPG expression existed in ACC, BRCA, COAD, ESCA, HNSC, KIRP, LGG, LIHC, LUSC, OV, SKCM, and UCEC (Supplementary Figure S7). Above all, these findings confirm that NCAPG has distinct patterns of expression in different cancer tissues.

### NCAPG Was Highly Expressed in NSCLC

**FIGURE 2:**
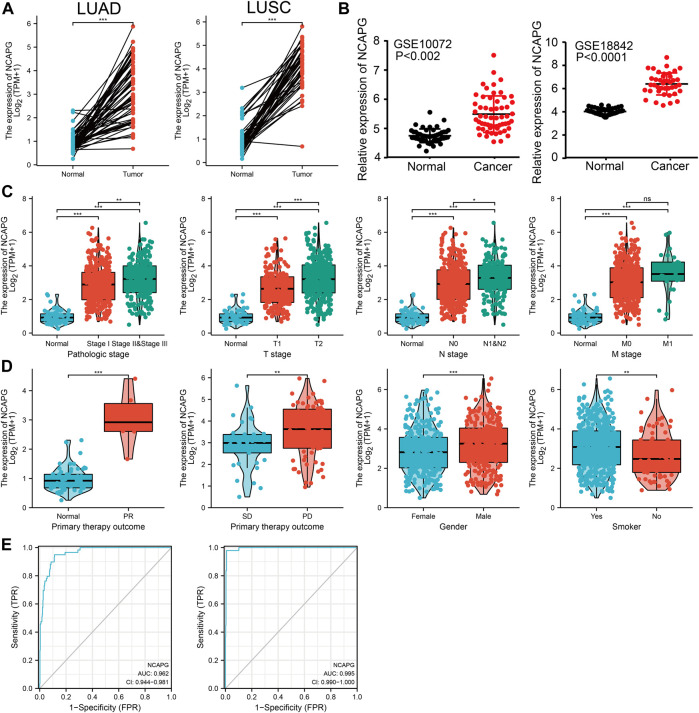
NCAPG was a high expression in NSCLC. **(A–D)** The expression of NCAPG in NSCLC was examined by TCGA and GEO databases. **(C,D)** NCAPG expression is associated with clinicopathological characteristics in NSCLC. **(E)** Receiver operating characteristic analysis (ROC) of NCAPG in NSCLC. (*p < 0.05, **p < 0.01, ***p < 0.001).

To elucidate the biological functions of NCAPG in NSCLC, we first examine the expression of NCAPG in NSCLC by using TCGA and GEO database, the results showed that the expression of NCAPG, but not of NCAPG2, was significantly up-regulated in NSCLC (Figures 2A, B), Notably, NCAPG expression was positively associated with poor clinical-pathological features, including the pathologic stage, TNM stage, primary therapy outcome, gender, and smoking status (Figures 2C, D).

**FIGURE 3:**
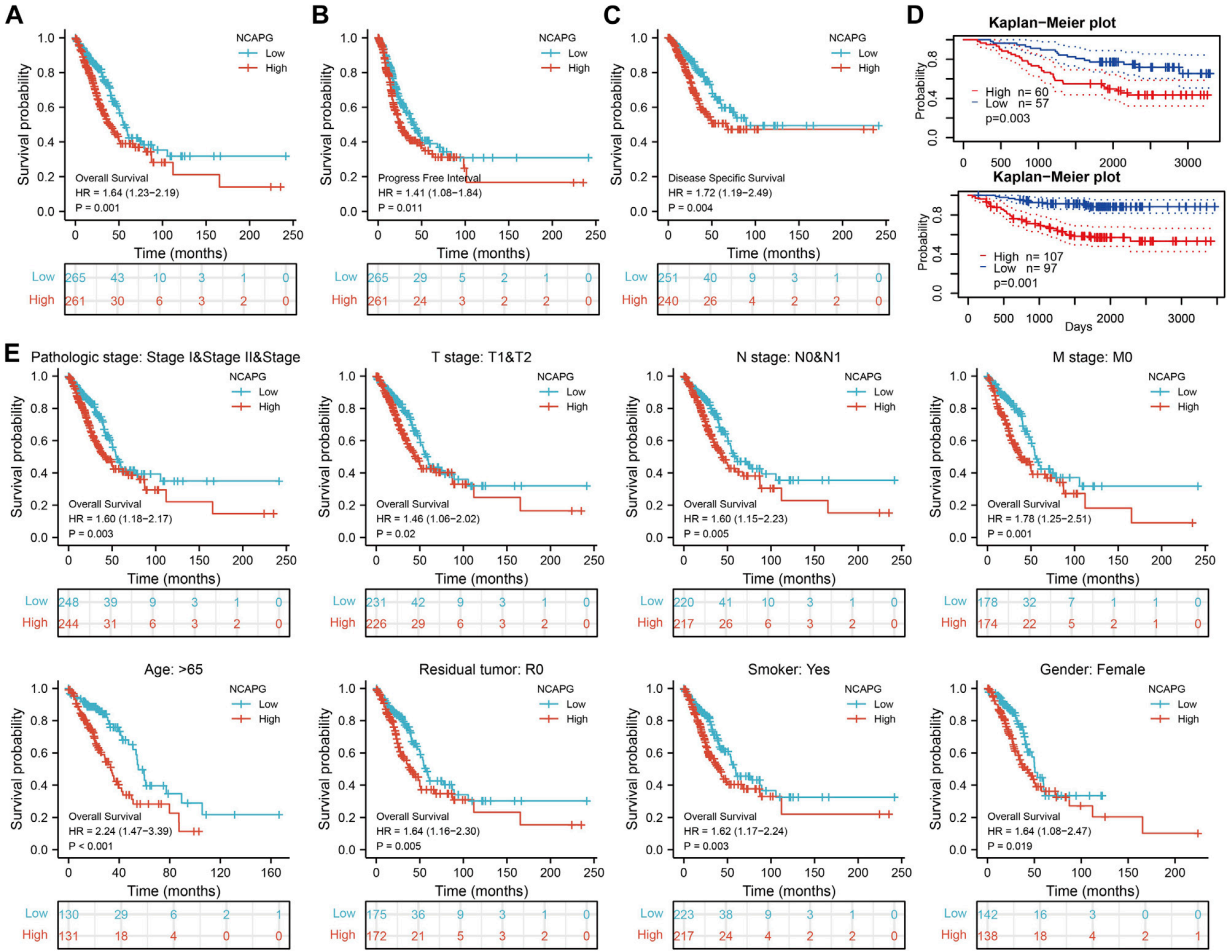
The prognosis of NCAPG in NSCLC. **(A–C)** The prognosis of NCAPG in NSCLC is examined by the TCGA database. **(D)** The prognosis of NCAPG in NSCLC is examined by the GEO database. **(E)** Distinct clinical outcomes based on NCAPG expression in NSCLC patient subgroups. *p < 0.05, **p < 0.01, ***p < 0.001.

We used logistics analysis to examine the correlation between NCAPG expression and clinicopathological characteristics. We found that NCAPG expression was positively correlated with the clinical stage including the TNM stage, pathologic stage, primary therapy outcome, gender, and smoker (Table 1). ROC curve analysis showed that the AUC for NCAPG in LUAD and LUSC patients was 0.963 and 0.995, respectively (Figure 2E). These findings indicated that the elevated expression level of NCAPG was correlated with poor pathobiology features of NSCLC patients. Given that NCAPG was significantly associated with poor pathobiology features in NSCLC patients, therefore, we further analyzed the prognosis value of NCAPG in NSCLC, the results showed that high expression of NCAPG was correlated with poor prognosis in NSCLC, this result was verified by GEO data (Figures 3A–D). The prognosis analysis for different subgroups showed that high expression of NCAPG was associated with poor prognosis (Figure 3E).

**TABLE 1:** Logistic regression analysis of correlation between clinical–pathological characteristics and NCAPG expression in NSCLC patients.

Characteristics	Total (n)	Odds ratio	*p* Value
T stage (T2 and T3 and T4 vs. T1)	532	2.128 (1.473–3.092)	<0.001
N stage (N1 and N2 and N3 vs. N0)	519	1.803 (1.245–2.624)	0.002
M stage (M1 vs. M0)	386	3.114 (1.283–8.715)	0.018
Pathologic stage (stage III and stage IV vs. stage I and stage II)	527	2.178 (1.414–3.399)	<0.001
Primary therapy outcome (PD vs. SD)	108	2.067 (0.921–4.695)	0.079
Gender (male vs. female)	535	1.905 (1.352–2.693)	<0.001
Age (>65 vs. ≤65 years)	516	0.768 (0.543–1.085)	0.135
Residual tumor (R1 and R2 vs. R0)	372	2.230 (0.809–7.130)	0.139
Smoker (yes vs. no)	521	1.751 (1.067–2.914)	0.028

**FIGURE 4:**
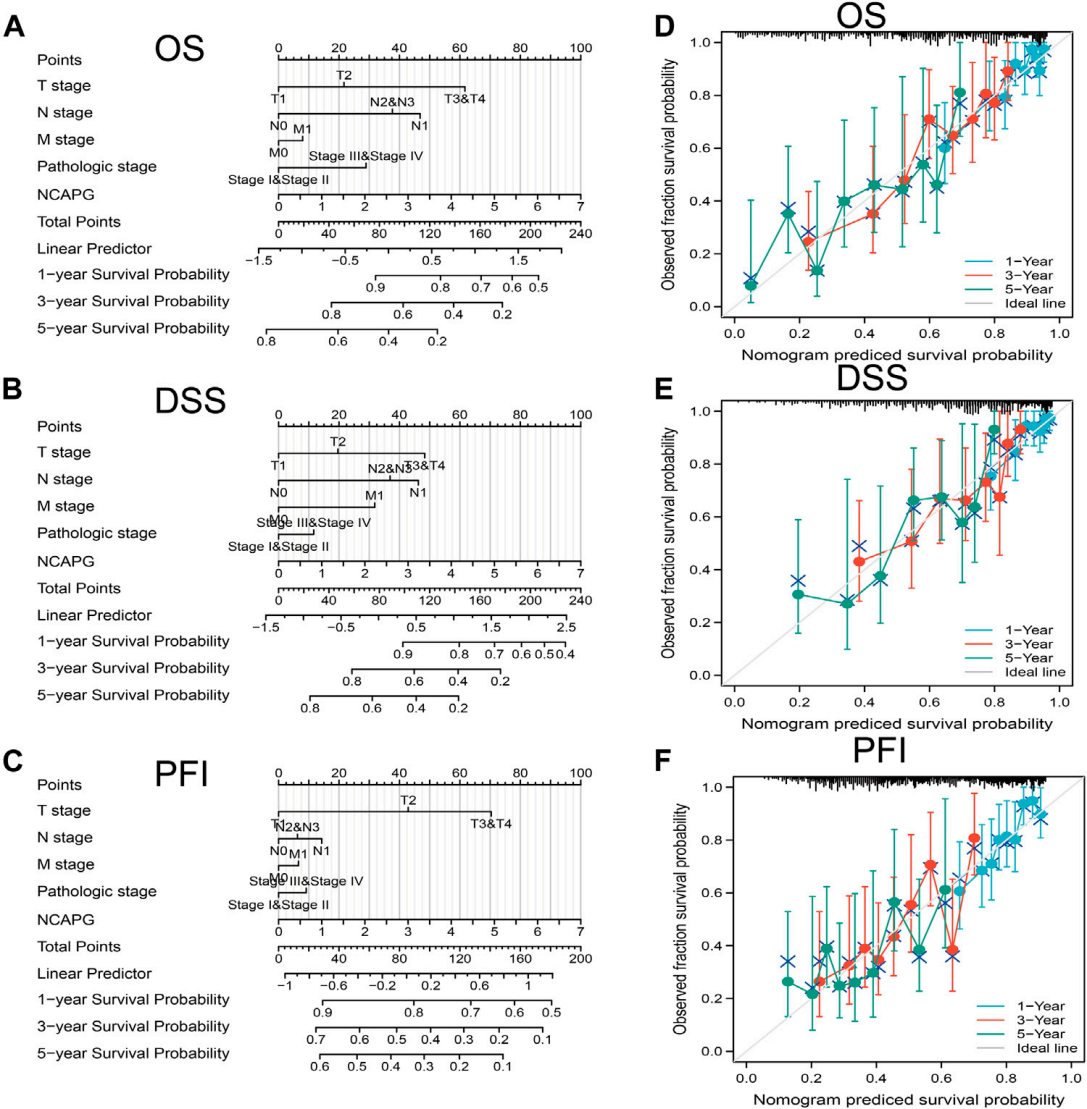
Nomogram for prediction of prognosis in NSCLC patients. Construction and validation of nomograms based on NCAPG expression. Shown are the nomograms constructed to establish NCAPG expression-based risk scoring models for 1-, 3-, and 5-year overall survival **(A)**, disease-specific survival **(B)**, and progression-free interval **(C)**. Calibration plots validate the efficiency of nomograms for overall survival **(D)**, disease-specific survival **(E)**, and progression-free interval **(F)**. OS, overall survival; FI, progression-free interval; DSS, disease-specific survival. *p < 0.05, **p < 0.01, ***p < 0.001.

All the statistically significant prognostic factors in each multivariate Cox regression analysis were utilized to construct a prognostic nomogram, the calibration curve was drawn to examine the efficiency of the nomogram. The clinical pathologic stage, TNM stage, and NCAPG expression, were included in the nomogram to predict OS, DSS, and progression-free interval (PFI); the c-index was OS: C-index: 0.709 (0.684–0.733), DSS: C-index: 0.710 (0.679–0.741), PFI: C-index: 0.645 (0.620–0.669) (Figures 4A–F). The calibration curves all presented desirable predictions of the three nomograms for the 1-, 3-, and 5-year clinical outcomes.

### Analysis of the Function of NCAPG in LUAD

**FIGURE 5:**
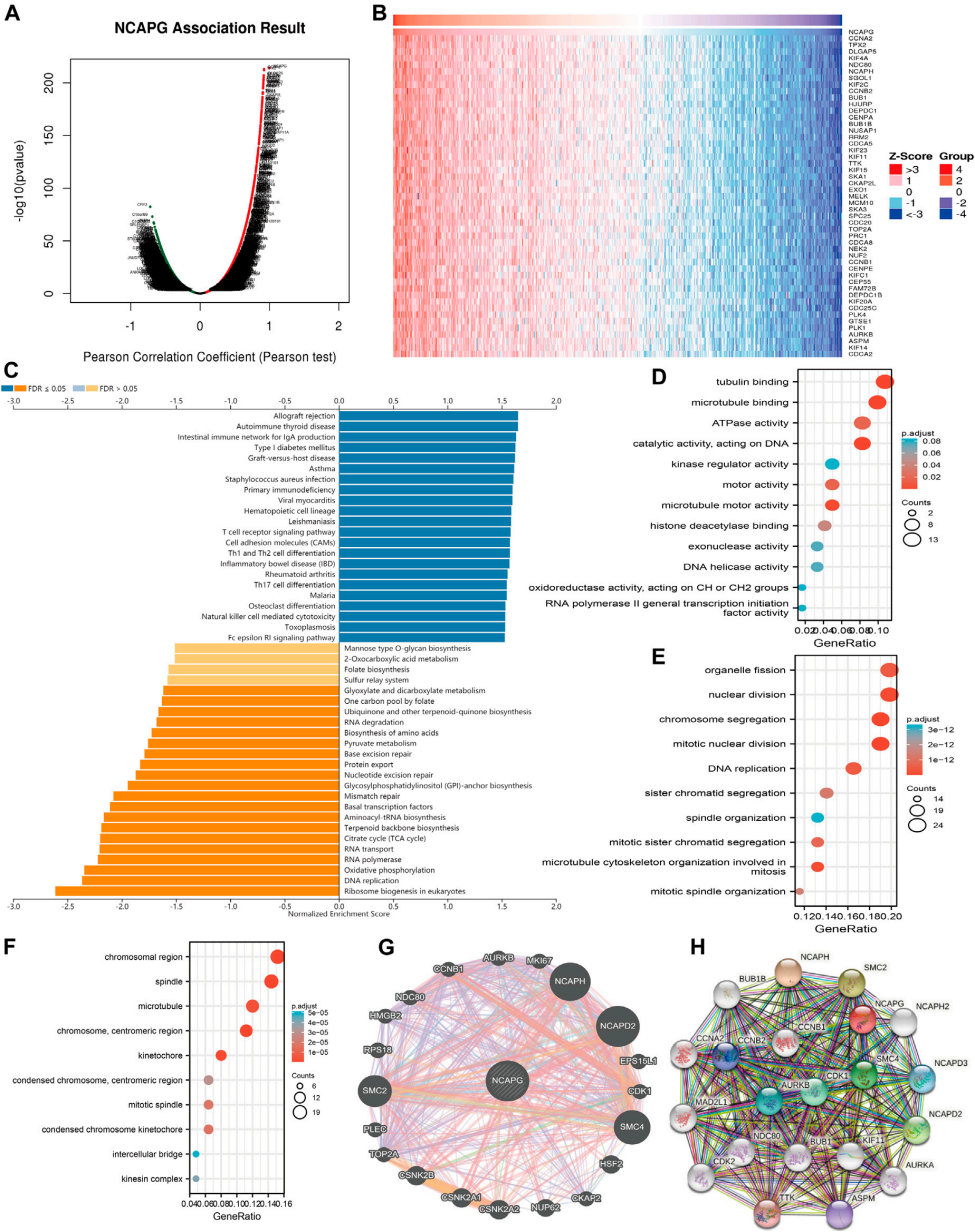
GO and KEGG enrichment analysis for NCAPG. **(A)** Volcano plot of genes differentially expressed in correlation with NCAPG. **(B)** Heat maps of genes positively correlated with NCAPG (top 50) **(C)** Analysis of the KEGG pathway of NCAPG in NSCLC by linkedomics. **(D–F)** GO analysis for NCAPG in NSCLC. **(G)** The gene interaction meshwork of NCAPG was constructed by using GeneMania. **(H)** Utilized the STRING to construction the protein interaction meshwork of NCAPG. *p < 0.05, **p < 0.01, ***p < 0.001.

After determining the prognostic value of NCAPG in LUAD, we next explored the functions of NCAPG in NSCLC progression. The function module of linked-omics was used to examine the NCAPG coexpression gene in TCGA-NSCLC cohort (Figure 5A); the genes that were most positively related to NCAPG are exhibited in the heat map (Figures 5B). KEGG pathway enrichment analysis showed that NCAPG-coexpressed genes mainly were involved in primary immunodeficiency, hematopoietic cell lineage, T-cell receptor signaling pathway, cell adhesion molecules, cell differentiation of Th1 and Th2, cell differentiation of Th17, natural killer cell–mediated cytotoxicity, and Fc epsilon RI signaling pathway (Figure 5C). These conclusions intensively indicated that NCAPG is involved in the regulation of the immune response in NSCLC.

For the GO term, NCAPG-coexpressed genes are mainly involved in the molecular function of microtubule-binding, tubulin binding, catalytic activity, acting on DNA, microtubule motor activity ATPase activity, motor activity, histone deacetylase binding, DNA helicase activity, exonuclease activity, and kinase regulator activity (Figure 5D). NCAPG-coexpressed genes are mainly involved in the biology process of mitotic nuclear division, chromosome segregation, nuclear division, microtubule cytoskeleton organization involved in mitosis, DNA replication, organelle fission, mitotic sister chromatid segregation, sister chromatid segregation, mitotic spindle organization, and spindle organization (Figure 5E). NCAPG-coexpressed genes are mainly involved in the cellular component of the chromosomal region, spindle, chromosome, centromeric region, kinetochore, microtubule, condensed chromosome kinetochore, mitotic spindle, condensed chromosome, centromeric region, kinesin complex, and intercellular bridge (Figure 5F). Furthermore, we used the Gene Mania database to analyze the gene interaction meshwork of NCAPG and the altered adjacent to genes. The results suggest the 20 constantly changing genes were closely correlated with NCAPG, including MKI87, NCAPH, NCAPD2, CDK1, HSF2, CCNB1, and AURKB (Figure 5G). Finally, we also used the STRING database to construct the PPI network of NCAPG (Figure 5H). We further analyzed the functions of this interaction gene with NCAPG; the result was suggestive of these genes mainly linked to the cell cycle and cell proliferation. Collectively, these findings emphasize the crucial role of NCAPG in regulating the progression of NSCLC.

### Analysis NCAPG-Related Signaling Pathways in LUAD by GSEA

**FIGURE 6:**
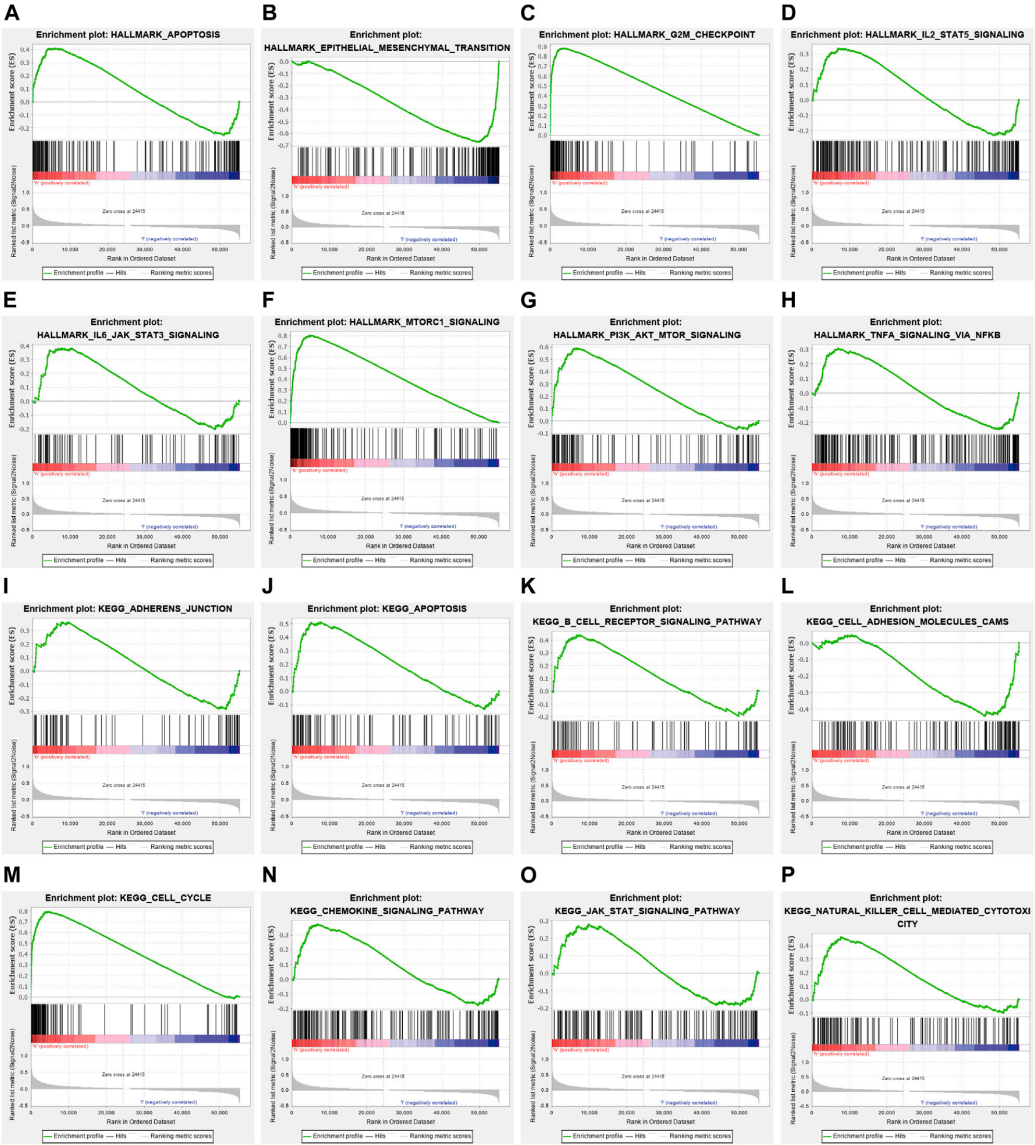
The signaling pathway for NCAPG enrichment by GSEA software. **(A–P)** The signaling pathway for NCAPG enrichment by GSEA software.

To examine the potential mechanisms influenced by NCAPG in LUAD, GSEA was conducted. The analysis result showed that NCAPG mainly participates in apoptosis, epithelial–mesenchymal transition, G2/M checkpoint, IL-2 STAT5 signal pathway, IL-6–JAK–STAT3 signal pathway, and MTORC1signaling pathway in LUAD (Figures 6A–H). Similarly, relating to the KEGG pathways, the enriched result proves that the NCAPG is mainly involved in B-cell receptor signal pathway and natural killer cell–mediated cytotoxicity signaling pathway (Figures 6I–P). The aforementioned findings intensively were indicative of NCAPG participation in lung cancer progression and immune response.

### NCAPG Expression Is Correlated With Immune Infiltration in NSCLC

**FIGURE 7:**
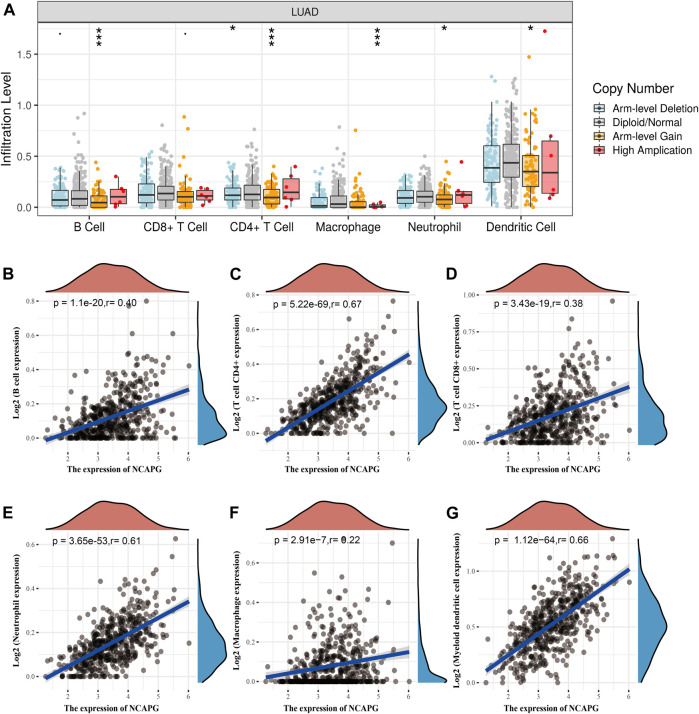
The correlation between NCAPG expression and immune infiltration in NSCLC. **(A)** The NCAPG gene copy numbers affect the infiltration level of different immune cells in LUAD. **(B–G)** NCAPG positively linked to the infiltration of different immune cells examined by TIMER databases. *p < 0.05, **p < 0.01, ***p < 0.001.

Tumor-infiltrating lymphocyte plays crucial roles in the prognosis of NSCLC (Fan et al., 2021; Zeng et al., 2021). First, we used TIMER online analysis tool to examine the relationship between the immune infiltration and the somatic copy number alterations of NCAPG in LUAD. Particularly, NCAPG CNV has memorable correlations with infiltrating levels of B cells, CD4^+^ T cells, macrophages, neutrophils, and dendritic cells (Figure 7A). Next, we further explored the correlation between NCAPG expression and the infiltration levels of different immune cells in TCGA-LUAD patients. NCAPG expression was weakly correlated with macrophages (*r* = 0.22, *p* = 2.91e-07), moderately with CD8^+^ T cells (*r* = 0.38, *p* = 3.43e-19) and B cells (*r* = 0.40, *p* = 1.1e-20), and intensively with neutrophils cells (*r* = 0.61, *p* = 3.65e-13), myeloid dendritic cells (*r* = 0.66, *p* = 1.12e-64), and CD4^+^ T cells (*r* = 0.67, *p* = 5.22e-69) (Figures 7B–G).

To further examine the functions of NCAPG in tumor immune, we used the TIMER database to explore the correlation between the NCAPG expression and immune cell–related markers in LUAD. The result indicated that NCAPG was markedly positively linked to different immune cells, containing B cells, CD8^+^ T cells, CD8^+^ T cells, M1 macrophage biomarkers, M2 macrophage biomarkers, neutrophil and dendritic cell–related markers in LUAD (Table 2).

**TABLE 2:** The correlation between NCAPG and gene markers of immune cells examined by TIMER database.

Description	Gene markers	LUAD			
		None		Purity	
		Cor	*p*	Cor	*p*
B cell	CD19	0.501	***	0.347	***
—	CD79A	0.415	***	0.275	***
T cell (general)	CD3D	0.65	***	0.633	***
—	CD3E	0.718	***	0.543	***
—	CD2	0.736	***	0.654	***
CD8^+^ T cell	CD8A	0.551	***	0.445	***
—	CD8B	0.444	***	0.348	***
Monocyte	CD86	0.581	***	0.475	***
—	CSF1R	0.62	***	0.533	***
TAM	CCL2	0.382	***	0.265	***
—	CD68	0.436	***	0.335	***
—	IL10	0.449	***	0.328	***
M1	IRF5	0.55	***	0.482	***
M2	CD163	0.449	***	0.342	***
—	VSIG4	0.397	***	0.304	***
—	MS4A4A	0.425	***	0.304	***
Neutrophils	CEACAM8	0.202	***	0.196	***
—	ITGAM	0.602	***	0.523	***
—	CCR7	0.704	***	0.622	***
Natural killer cell	KIR2DL1	0.173	***	0.109	***
—	KIR2DL3	0.239	***	0.163	***
—	KIR2DL4	0.261	***	0.178	***
—	KIR3DL1	0.207	***	0.136	***
—	KIR3DL2	0.295	***	0.22	***
—	KIR2DS4	0.209	***	0.134	***
Dendritic cell	HLA-DPB1	0.621	***	0.54	***
—	HLA-DQB1	0.486	***	0.391	***
—	HLA-DRA	0.567	***	0.472	***
—	HLA-DPA1	0.602	***	0.527	***
—	CD1C	0.46	***	0.385	***
—	NRP1	0.267	***	0.238	***
—	ITGAX	0.644	***	0.559	***
Th1	TBX21	0.689	***	0.628	***
—	STAT4	0.615	***	0.51	***
—	STAT1	0.474	***	0.385	***
—	TNF	0.549	***	0.448	***
—	IFNG	0.486	***	0.4	***
Th1-like	HAVCR2	0.578	***	0.468	***
—	CXCR3	0.696	***	0.62	***
—	BHLHE40	0.235	***	0.204	***
—	CD4	0.673	***	0.582	***
Th2	STAT6	0.287	***	0.337	***
—	STAT5A	0.671	***	0.597	***
Treg	FOXP3	0.725	***	0.648	***
—	CCR8	0.658	***	0.578	***
—	TGFB1	0.287	***	0.409	***
Effector Treg T cells	TNFRSF9	0.684	***	0.576	***
—	FGFBP2	0.182	***	0.114	***
—	FCGR3A	0.458	***	0.35	***
Effector T cells	CCR7	0.704	***	0.622	***
—	SELL	0.662	***	0.568	***
Naive T cells	GZMK	0.618	***	0.519	***
—	CD69	0.569	***	0.47	***
Effector memory T cells	CXCR6	0.624	***	0.52	***
—	MYADM	0.314	***	0.227	***
Resident memory T cells	IL7R	0.584	***	0.475	***
—	HAVCR2	0.578	***	0.468	***
—	LAG3	0.551	***	0.469	***
—	CXCL13	0.502	***	0.371	***
Memory T cells	LAYN	0.336	***	0.2	***

*p < 0.05, **p < 0.01, ***p < 0.001.

Immune checkpoints play a significant role in cancer immune evasion (Qu et al., 2021). In consideration of the crucial roles of NCAPG in LUAD, the correlation of NCAPG with immune checkpoints related genes was assessed. NCAPG expression was significantly positively linked to CD274 (*r* = 0.566, *p* = 4.79e-45), CTLA4 (*r* = 0.716, *p* = 4.9e-82), PDCD1 (*r* = 0.659, *p* = 1.84e-65), TIGIT (*r* = 0.738, *p* = 1.06e-89), PDCD1LG2 (*r* = 0.596, *p* = 7.58e-51), HAVCR2 (*r* = 0.578, *p* = 3.21e-47), LAG3 (*r* = 0.551, *p* = 3.39e-42), and SIGLEC15 (*r* = 0.327, *p* = 2.74e-14) in LUAD (Supplementary Figures S8A–S8H). In addition, we also found significant correlations of NCAPG with immune infiltration and immune inhibitors across human heterogeneous cancers (Supplementary Figures S8I–S8J). These results indicated that NCAPG might play significant roles in the regulation of immune infiltration of LUAD.

### Analysis of the Prognosis of the Immune Infiltration and NCAPG Expression in LUAD

**FIGURE 8:**
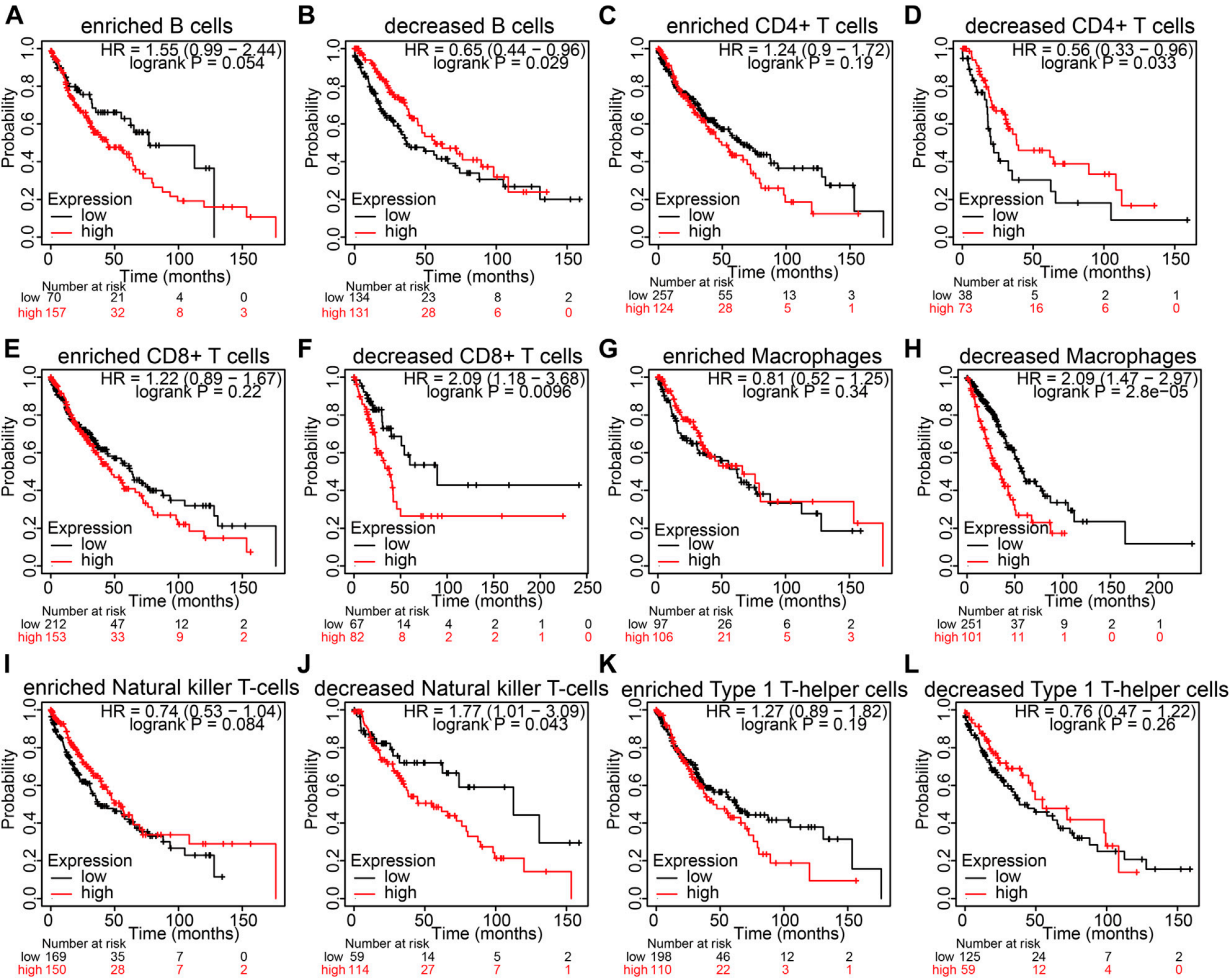
The prognosis analysis based on the expression of NCAPG in different immune cell subgroups in NSCLC. **(A–L)** Correlation between NCAPG expression and OS in different immune cell infiltration groups in LUAD patients. *p < 0.05, **p < 0.01, ***p < 0.001.

NCAPG expression is markedly related to immune infiltration and unfavorable prognosis in LUAD (Fan et al., 2021). Next, we examined whether NCAPG expression affects the prognosis of LUAD through immune infiltrate. We used the Kaplan–Meier plotter analysis of the prognosis of NCAPG in LUAD in different related subgroups of immune cells. The analysis results suggest elevated NCAPG expression and reduced level of B cells, CD4^+^ memory T cells, CD8^+^ memory T cells, macrophages, and natural killer T cells, which had an unfavorable prognosis observed in the LUAD patient (Figures 8A–L). The prognosis of LUAD was of no relevance in other immune cell groups. These findings suggest that NCAPG may affect the survival state of LUAD patients, partially owing to immune infiltrate.

### Depletion of NCAPG Inhibits NSCLC Progression

**FIGURE 9:**
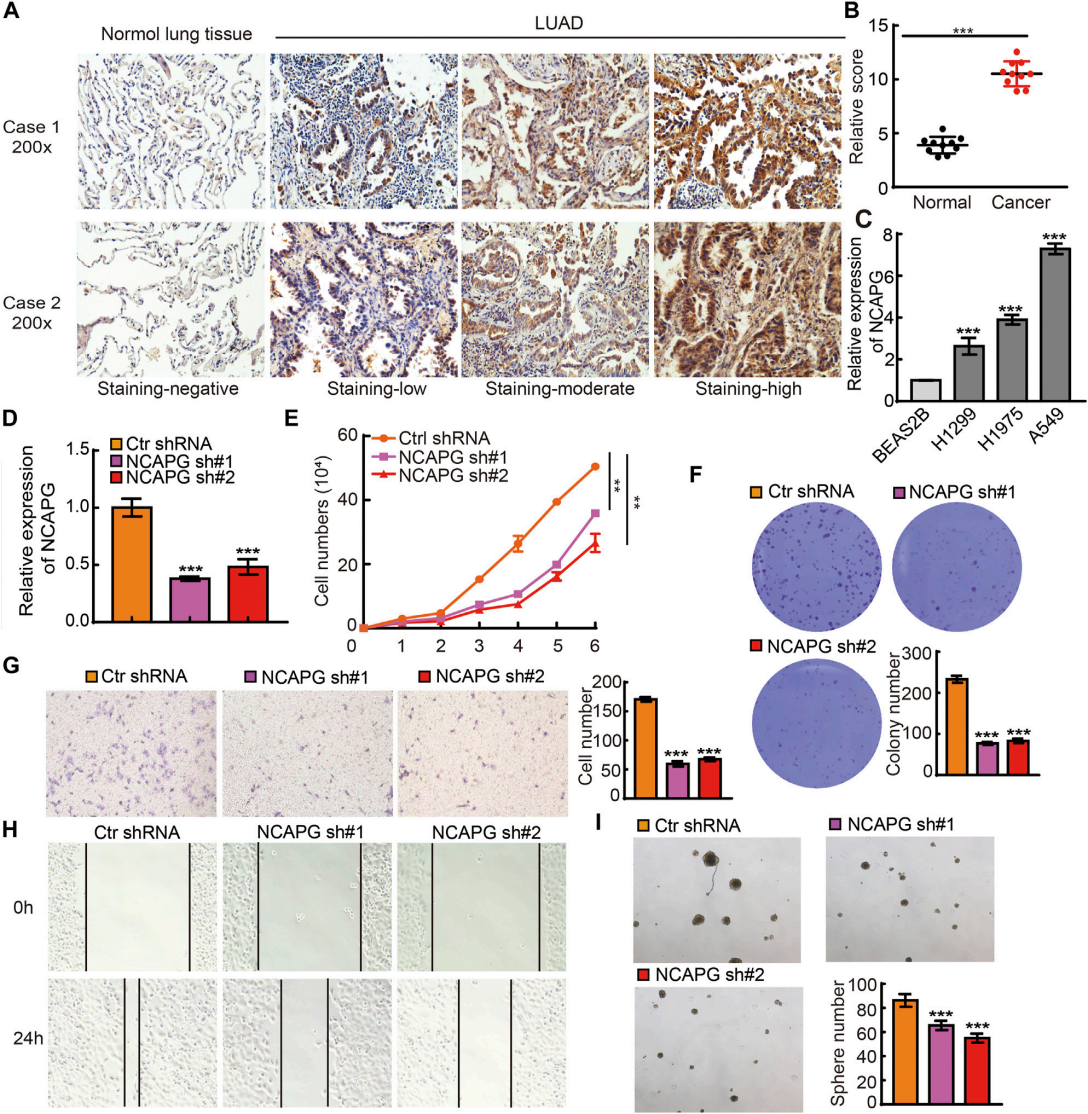
NCAPG silencing on cell on proliferation, migration, and self-renewal ability of NSCLC Cell. **(A)** IHC analysis of NCAPG in lung cancer tissues and normal tissues. **(B)** IHC scores statistical analysis of NCAPG in lung cancer tissues and normal tissues. **(C)** The expression of NCAPG in NSCLC cell lines was examined by qRT-PCR assay. **(D)** The establishment of NCAPG knockdown in A549 cell lines and verified by real-time qPCR **(E,F)** Growth curve and clone formation assays were utilized to detect silencing of NCAPG on the growth of A549 cell. **(G,H)** Transwell and wound healing assays were used to detect silencing of NCAPG on the migration of A549 cells. **(I)** A tumorsphere experiment was used to detect silencing of NCAPG on the self-renewal of A549 cells. *p < 0.05, **p < 0.01, ***p < 0.001.

To examine the protein expression of NCAPG in NSCLC, we utilized an IHC assay to examine the expression of NCAPG in lung cancer. The results verify that the elevated expression of NCAPG was observed in lung cancer than the normal tissues (Figures 9A, B). Next, we examined the mRNA expression of NCAPG in BEAS2B and NSCLC cell lines. The result showed that NCAPG expressions are significantly elevated in all NSCLC cells, highest expression NCAPG observe in A549 (Figure 9C). Owing to NCAPG being highly expressed in NSCLC cells lines, we speculated that NCAPG might play a crucial role in the progression of NSCLC. We then selected the A549 cell for further studies. We inhibited the NCAPG mRNA expression using two independent lentiviral shRNAs targeting different regions of NCAPG. The efficiency and specificity of NCAPG knockdown in A549 cells were verified by quantitative reverse transcriptase (qRT)–PCR (Figure 9D). Growth curve and colony formation assays were carried out to evaluate cell proliferation, and the results showed that NCAPG silencing notably inhibited the proliferation of A549 cells (Figures 9E, F). Transwell and wound healing experiments showed that NCAPG knockdown reduced the migration of A549 cells (Figures 9G, H). Tumorsphere assay showed that NCAPG knockdown reduced the self-renewal of GSCs (Figures 9I). Collectively, these findings indicate NCAPG exerts an oncogenic role in NSCLC cells.

### Identification of miR-214-3p as the Direct Binding miRNA of NCAPG in LUAD

**FIGURE 10:**
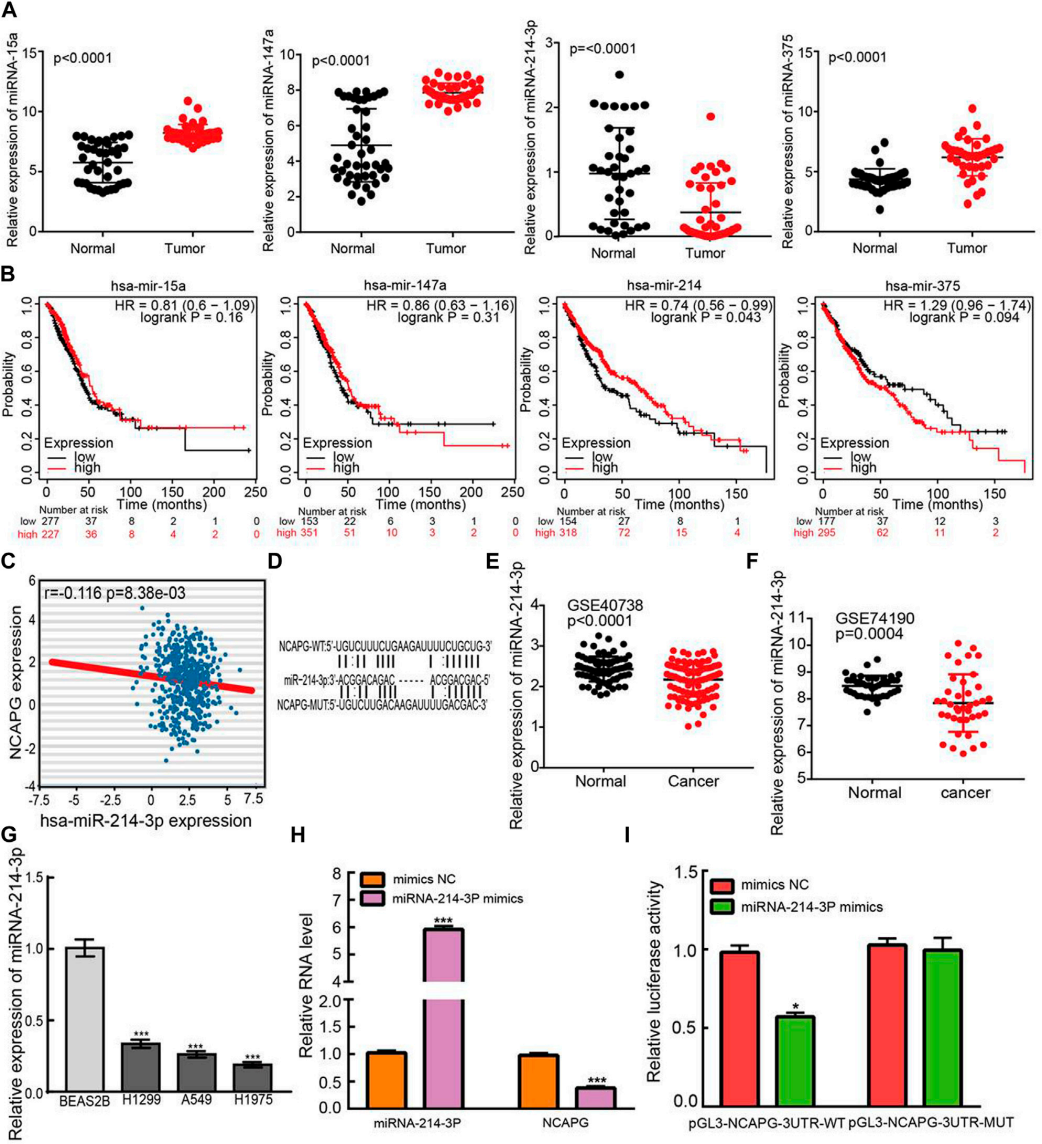
Identification of upstream miRNAs of NCAPG in LUAD. **(A)** The expression of miRNA-15a, miRNA-147a, miRNA-375, and miRNA-214-3p in TCGA-LUAD. **(B)** The prognostic values of miRNA-15a, miRNA-147a, miRNA-375, and miRNA-214-3p in TCGA-LUAD. **(C)** The correlation between expression of miRNA-214-3p and NCAPG in LUAD. **(D)** The potential binding sites of miR-214-3p and NCAPG. **(E)** The expression of miRNA-214-3p in NSCLC was examined by the GEO database. **(F)** The expression of miRNA-214-3p in NSCLC determined by GEO database **(G)** The expression of miRNA-214-3p in NSCLC cell lines. **(H)** The expression of NCAPG in A549 cell after overexpression of miRNA-214-3p examined by the qRT-PCR assay. **(I)** Relative luciferase activities of wild type (WT) and mutated (MUT) NCAPG–3′ UTR reporter plasmid in A549 cells cotransfected with miRNA-214-3p mimics. *p < 0.05, **p < 0.01, ***p < 0.001.

To uncover whether NCAPG was regulated by miRNAs, we predicted the potential miRNAs that bind with NCAPG by using the RNA22 (Loher and Rigoutsos, 2012), miRmap (Goossens et al., 2019), DIANA-microT web server v5.0 (Paraskevopoulou et al., 2013), miRanda (Rost and McGregor, 2012), and TargetScan (Agarwal et al., 2015). Eventually, only four miRNAs, including miRNA-15a, miRNA-147a, miRNA-375, and miRNA-214-3p, were commonly predicted. Next, we assessed their expression levels and prognostic values in NSCLC using TCGA-LUAD data (Figures 10A, B). According to the ceRNA theory, the miRNA could bind with 3′ UTR of mRNA and inhibit the expression of mRNA; thus, the expression of miRNA and mRNA should have a negative correlation pattern. We performed the expression correlation between NCAPG and miRNA-214-3p, the result demonstrated that NCAPG was significantly negatively correlated with miRNA-214-3p in LUAD (Figure 10C). In addition, Among the four miRNAs, only miR-214-3p was significantly decreased in LUAD than a control group. Survival analysis demonstrated that the elevated miR-214-3p expression has a favorable prognosis in LUAD. By integration of the expression analysis, correlation analysis, and survival analysis, we supposed that miR-214-3p was the most potential miRNA that may be potentially bound to NCAPG, the potential binding site between the miRNA-214-3p and NCAPG 3′ UTR were predicted base-pairing with miRNA-214-3p by starBase (Figure 10D). Next, we analyzed the expressions of miRNA-214-3p in whole blood of human lung cancer and lung cancer by using the GEO database (Patnaik et al., 2017); these data also revealed miRNA-214-3p expressions are down-regulated in the whole blood of human lung cancer and lung cancer (Figures 10E,F). Finally, we also analyzed the expressions of miRNA-214-3p in normal human bronchial epithelial cells and NSCLC cell lines. Consistent with our findings in TCGA, we found that miRNA-214-3p expressions are down-regulated in all NSCLC cell lines (Figure 10G). To investigate the effects of miRNA-214-3p on the expression of NCAPG, we conducted overexpression analysis. Overexpressing miRNA-214-3p significantly reduced the mRNA expressions of NCAPG in A549 cells (Figure 10H). The luciferase assay showed that transfection of miRNA-214-3p mimics significantly the reduced relative luciferase activity of NCAPG-3UTR-WT–treated lung cancer cells, but did not affect that of NCAPG-3UTR-MUT–treated lung cancer cells (Figure 10I). Collectively, these data imply that miRNA-214-3p might target the NCAPG and plays pivotal roles in the regulation of NCAPG expression in NSCLC.

### Identification of TYMSOS as Upstream lncRNA of miRNA-214-3p in LUAD

**FIGURE 11:**
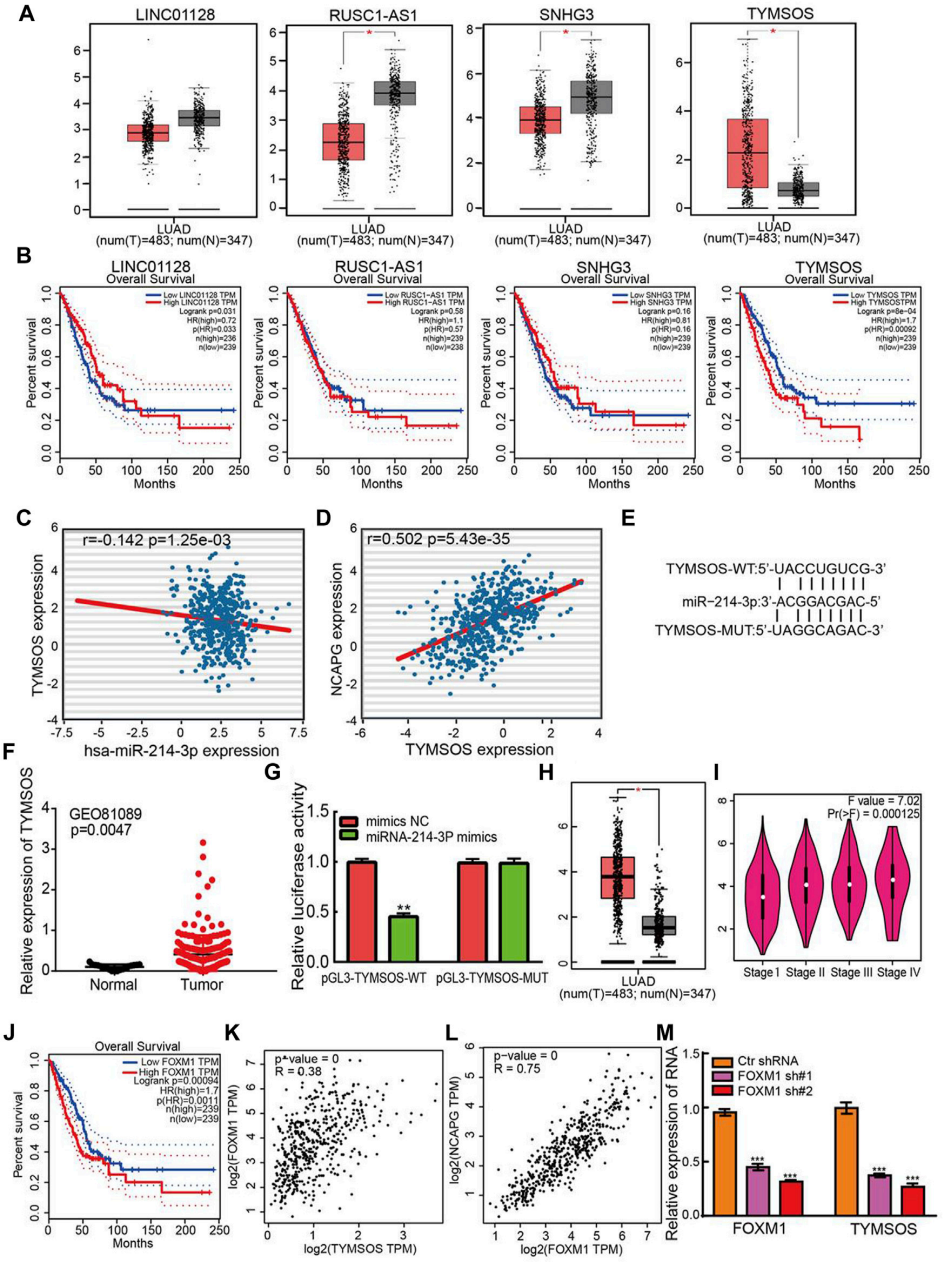
Identification of upstream lncRNAs of miRNA-214-3p in LUAD. **(A)** The expression of LINC01228, RUSC1-AS1, SNHG3, and TYMSOS in TCGA-LUAD. **(B)** The prognostic values of LINC01228, RUSC1-AS1, SNHG3, and TYMSOS in TCGA-LUAD. **(C)** The correlation between expression of miRNA-214-3p and TYMSOS in LUAD. **(D)** The correlation between expression of NCAPG and TYMSOS in LUAD. **(E)** The potential binding sites of miR-214-3p and TYMSOS. **(F)** The expression of TYMSOS in GSE81089 and control normal samples determined by GEO database. **(G)** Relative luciferase activities of wild-type (WT) and mutated (MUT) THTMSOS reporter plasmid in A549 cells cotransfected with miRNA-214-3p mimics. **(H)** The expression of FOXM1 in LUAD was examined by using the GEPIA database. **(I)** The pathological stage analysis for FOXM1 in LUAD was determined by the GEPIA database. **(J)** The overall survival analysis for FOXM1 in LUAD was examined by using the GEPIA database. **(K)** The correlation between expression of FOXM1 and TYMSOS in TCGA-LUAD. **(L)** The correlation between expression of FOXM1 and NCAPG in TCGA-LUAD. **(M)** TYMSOS expression in A549 cell after knockdown FOXM1 was examined by real-time PCR. *p < 0.05, **p < 0.01, ***p < 0.001.

Diverse regulatory mechanisms of gene expression have been well established in the NSCLC progression. Emerging evidence has demonstrated that lncRNAs play an indispensable role in regulating gene expression. Next, the upstream lncRNAs of miRNA-214-3p were predicted using the starBase database and DIANA-LncBase v3 (Karagkouni et al., 2020). There are four lncRNAs were predicted, including LINC01228, RUSC1-AS1, SNHG3, and TYMSOS. Identically, the expression levels of and prognostic values of lncRNAs in LUAD were determined by using TCGA-LUAD data. The analysis results confirmed that LINC01228, RUSC1-AS1, and SNHG3 were down-regulated in NSCLC, on the contrary, TYMSOS was significantly up-regulation in NSCLC and high expression of TYMSOS was correlated with unfavorable prognosis in NSCLC (Figures 11A,B). In the light of the competitive endogenous RNAs theory, lncRNAs able to up-regulation the mRNA level via reducing the expression of miRNAs. Therefore, the elevated expression of lncRNA should decrease the level of miRNA, and positive correlation between mRNA. Among all the four lncRNAs, only TYMSOS was negatively correlated with miRNA-214-3p in LUAD (Figure 11C), and TYMSOS was positively correlated with NCAPG in LUAD (Figure 11D). The target sites in the miRNA-214-3p and TYMSOS were predicted to pair with miRNA-214-3p by TarBase (Figure 11E). Moreover, the TYMSOS was markedly elevated in LUAD matched to normal controls by analysis of the GEO databases (Mezheyeuski et al., 2018) (Figure 11F). Finally, to further confirm the interaction between TYMSOS and miRNA-214-3p, the luciferase assay was performed, the luciferase assay indicated that transfection of miRNA-214-3p mimics significantly decreased the relative luciferase activity of TYMSOS-WT–treated lung cancer cells, but did not affect that of TYMSOS-MUT–treated lung cancer cells (Figure 11G). Taken together, the data suggest that TYMSOS acted as a sponge for miRNA-214-3p in NSCLC cells.

To explore the up-regulation mechanism of TYMSOS in LUAD by using the JASPAR, PROMO, ConTra v3, and UCSC databases. We identified transcription factors that may regulate the expression of TYMSOS. Data indicated that FOXM1 was the most qualified one. We examined the mRNA expression of FOXM1 by using the GEPIA database. FOXM1expression was substantially elevated in LUAD (Figure 11H), the expression of FOXM1 was significantly elevated in LUAD patients than normal controls based on tumor grade (Figure 11I). Up-regulation of FOXM1expression is associated with the poor prognosis in lung cancer patients based on the GEPIA database (Figure 11J). We examined the expression relationship between FOXM1 and TYMSOS in TCGA-LUAD. The expression of FOXM1 was markedly linked to the expression of TYMSOS and NCAPG in LUAD (Figures 11K, L). Next, the knockdown plasmids of FOXM1 were constructed and respectively transfected into the A549 cell. Then, qPCR assays revealed that knockdown of FOXM1 can markedly reduce the expression of TYMSOS levels (Figure 11M). Collectively, these findings indicated that FOXM1 may be a potential transcriptional activator of TYMSOS.

### Depletion of TYMSOS Inhibits Cell Proliferation, Migration, and Self-Renewal Abilities of NSCLC Cells

**FIGURE 12:**
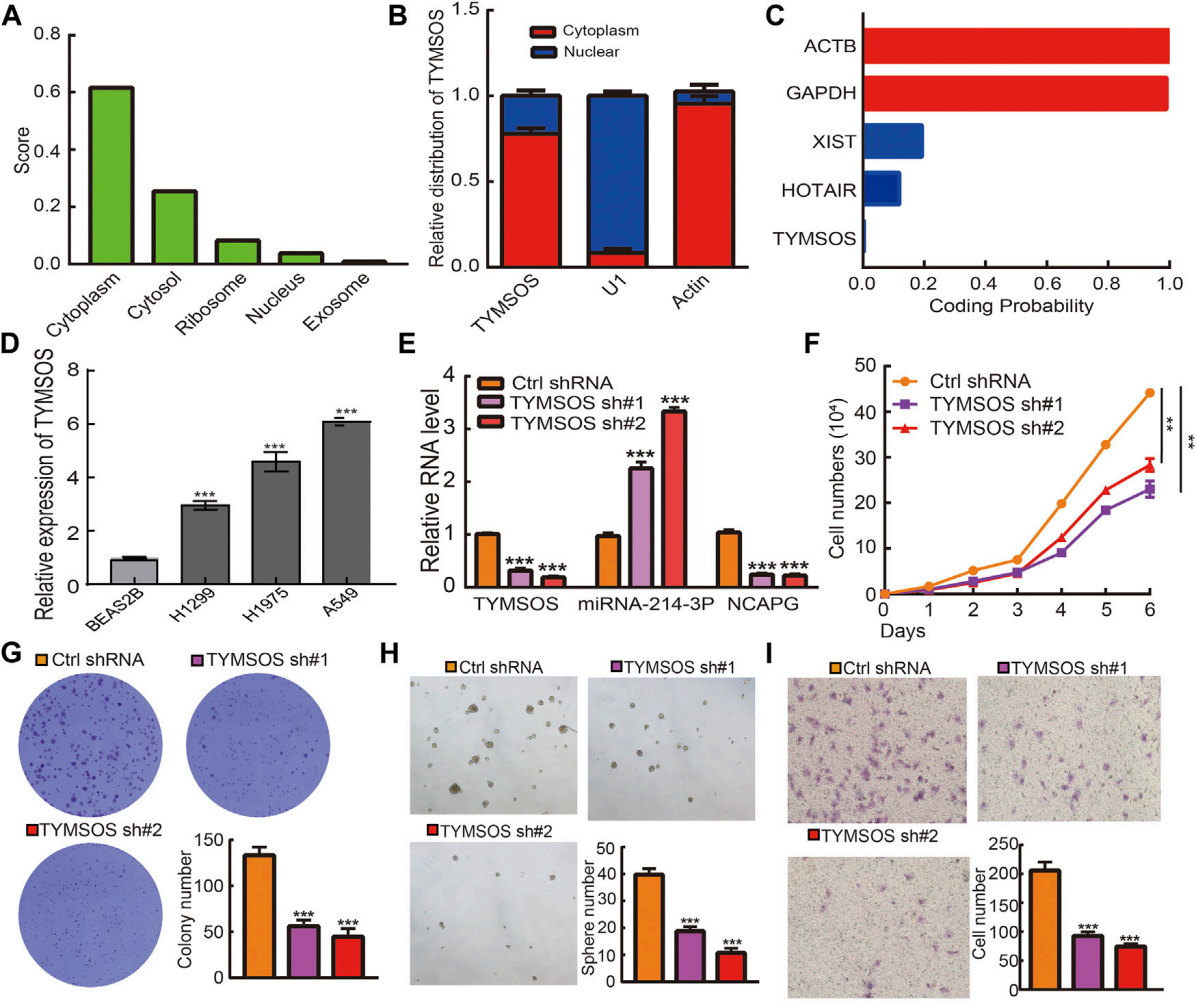
TYMSOS silencing on cell on proliferation, migration, and self-renewal ability of NSCLC cell. **(A)** The cellular localization for TYMSOS was predicted by using lncLocator. **(B)** The cellular localization for TYMSOS was examined by nuclear and cytoplasmic extraction experiments. **(C)** The coding ability for TYMSOS was predicted by using Coding Potential Calculator. **(D)** The expression of TYMSOS in NSCLC cells lines was examined by qRT-PCR assay. **(E)** The expression of miRNA-214-3p and NCAPG in A549 cells after knockdown of TYMSOS was examined by qRT-PCR assay. **(F,G)** The growth curve and clone formation assays were used to detect the silencing of TYMSOS on the growth of A549 cells. **(H)** The tumorsphere assay was utilized to detect silencing of TYMSOS on the self-renewal of the A549 cell. **(I)** The Transwell assay was utilized to detect silencing of TYMSOS on the migration of A549 cells. *p < 0.05, **p < 0.01, ***p < 0.001.

The underlying mechanisms of TYMSOS were determined by cellular localization. The subcellular localization of TYMSOS used the lncLocator and by performing a nuclear/cytoplasmic separate experiment. The results indicated that TYMSOS was mainly located in the cytoplasm of A549 cells (Figures 12A,B). We also analyzed the coding potential of TYMSOS by performing the coding potential calculator, the results showed that TYMSOS does not possess the protein-coding ability (Figure 12C). We first examined the expression of TYMSOS in NSCLC cells lines; the analysis results indicated that TYMSOS was significantly up-regulated in NSCLC cells (Figure 12D). To explore the biological function of TYMSOS in lung cancer cells, the TYMSOS knockdown plasmid was transfected into the A549 cell. The results indicated that the expression of NCAPG and miRNA-214-3p were decreased and increased, respectively, after the knockdown of TYMSOS in A549 cells (Figure 12E). The growth curve and colony formation experiment showed that TYMSOS knockdown significantly inhibits cell proliferation (Figures 12F,G). To estimate the significance of TYMSOS in NSCLC progression, migration and self-renewal of A549 cells were investigated after the knockdown of TYMSOS. The tumorsphere assays indicated that the self-renewal ability of A549 cells was significantly suppressed by down-regulation of TYMSOS (Figure 12H). The Transwell assays indicated that the migratory ability of A549 cells was significantly suppressed by the down-regulation of TYMSOS (Figure 12I). These findings indicated that TYMSOS functioned as an oncogenic lncRNA in promoting cell growth, metastasis, and self-renewal of NSCLC cells. These data suggest that the TYMSOS/miRNA-214-3p/NCAPG axis plays a significant role in the progression of lung cancer.

## Discussion

According to the global cancer statistics in 2020, lung cancer results in the highest morbidity rates and brought huge economic burden worldwide (Sung et al., 2021). Despite there being many different treatments, including immunotherapy, more than half of the patients are diagnosed at an advanced stage, and the survival rate of patients is very low (Hirsch et al., 2017). The initiation and progression of lung cancer are an exceedingly complicated process that involves genetic mutations, tumor microenvironment, and the dysregulation of epigenetic pathways (Govindan et al., 2012; Duruisseaux and Esteller, 2018; Shi et al., 2019). Concurrently, epigenetic changes in lung cancer such as histone modifications (Chen et al., 2018), DNA methylation (Hulbert et al., 2017) and noncoding RNAs (ncRNAs) (Li et al., 2019) have been extensively studied. The RNAs mainly include snRNAs, snoRNAs, piRNAs, tRNAs, and ribosomal RNAs, miRNAs, lncRNAs, circular RNAs (Esteller, 2011; Hombach and Kretz, 2016; Li et al., 2018). Accumulating evidence has revealed that numerous ncRNAs are dysregulated and involved in lung cancer initiation and progression (Chen et al., 2016; Wang et al., 2018; Iqbal et al., 2019). Many studies demonstrate that miRNAs and lncRNAs are involved in the pathogenesis of lung cancer. For example, lncRNA H19 the elevated cell growth and metastasis ability by reducing the level of miR-200 (Zhao et al., 2019). LncRNA TUC338 promotes invasion of lung cancer by activating the MAPK pathway (Zhang et al., 2018).

NCAPG plays key roles in mitotic chromosome condensation and cancer progression (Sun et al., 2020). It has also been reported that NCAPG serves as a prognostic biomarker in HCC (Xiao et al., 2020). Combining bioinformatics analyses and biological function validation, our study provides evidence that NCAPG was elevated in lung cancer, and its high expression was correlated with poor clinical-pathological features, including the pathologic stage, TNM stage, primary therapy outcome, gender, and smoking status. Finally, OS analyses showed that the up-regulated expression of NCAPG was associated with a poorer survival state in NSCLC patients. Aberrant expression of NCAPG has been elucidated in various human cancers. For example, NCAPG exerted oncogenic functions in hepatocellular carcinoma via regulating PI3K/AKT signaling (Gong et al., 2019). Furthermore, NCAPG has been reported to inhibit cardia adenocarcinoma apoptosis and promote epithelial–mesenchymal transition through the Wnt/β-catenin signaling pathway (Zhang et al., 2021). All these studies indicated that NCAPG might exert a pivotal function in human cancers. In our study, we observed increased NCAPG in NSCLC, which was also associated with a poor prognosis. Similarly, high expression of NCAPG is associated with poor prognosis in ovarian cancer (Xu et al., 2020b).

For the function of NCAPG in NSCLC, we performed the GSEA and KEGG pathway enrichment analyses in TCGA-LUAD; the results indicated that NCAPG participates in the immune system in LUAD. It has been well recognized that the tumor microenvironment is able to affect the productiveness of immunotherapy and involved in the process of cancer (Fridman et al., 2017; Jarosz-Biej et al., 2019; McLaughlin et al., 2020). It has been demonstrated that immune modulators play crucial roles in tumor cell evasion of immune surveillance (Xue et al., 2017). Studies have confirmed that the increased immune checkpoints such as PD-L1, CTLA-4, TIM-3, and LAG3 in glioma help tumor immune evasion, leading to T-cell dysfunction (Xue et al., 2017; Woroniecka et al., 2018). Here, we take the lead to review that up-regulation of NCAPG was positively correlated with the immune cell infiltration in lung cancer. Moreover, we also found that the NCAPG expression was not only significantly positively associated with various immune cell markers but also positively correlated with the expression of immune checkpoints–related genes. More significantly, we showed that NCAPG expression could affect the OS state of LUAD patients through immune infiltration. These results suggest that NCAPG could as an effective immunotherapy target in LUAD. However, the crucial functions of NCAPG in the tumor-immune regulation still need further research.

A growing number of studies have reported that lncRNA plays an important role in gene expression regulation (Zhang et al., 2020). To figure out the upstream regulatory microRNA about NCAPG, we used different databases to predict potential microRNAs that may be linked to NCAPG. After performing comprehensive analysis and functional experiments, miRNA-214-3p was chosen as miRNA that may be binding to the NCAPG. Furthermore, previous findings as well indicated that miRNA-214-3p played inhibitory roles in tumor cell proliferation (Li et al., 2020). These results indicate that miRNA-214-3p could act as an upstream regulatory miRNA of NCAPG in LUAD. Through conducting lncRNA molecular characteristics, expression, survival, and correlation analysis, one of the most potential elevated lncRNA, TYMSOS, was identified. It has been reported to function as oncogenes in gastric cancer (Gu et al., 2021). For instance, TYMSOS through binding the miR-4739 and resulting in the elevated expression of ZNF703 gives rise to the progression of gastric cancer (Gu et al., 2021). In this study, we found that the TYMSOS and NCAPG expression was markedly elevated in LUAD matched to the control group, and the elevated expression of TYMSOS and NCAPG was associated with the poor outcome by survival analysis. On the contrary, miR-214-3p expression was reduced in LUAD, and the elevated miR-214-3p expression indicated better OS prognosis in LUAD. In addition, we also identified FOXM1 as a potential transcriptional activator of TYMSOS. We also performed functional assay to verify the function of TYMSOS and NCAPG in NSCLC; the results verified that knockdown of TYMSOS and NCAPG significantly inhibited the cell proliferation, migration, and self-renewal abilities of NSCLC cells. We also confirm the interaction between TYMSOS, miRNA-214-3p, and NCAPG. Although various molecular mechanisms can lead to increased gene expression, such as the DNA methylation, regulation of transcription factors, and gene copy number amplification, lncRNA/miRNA axis is one of the main regulatory mechanisms of gene expression.

**FIGURE 13:**
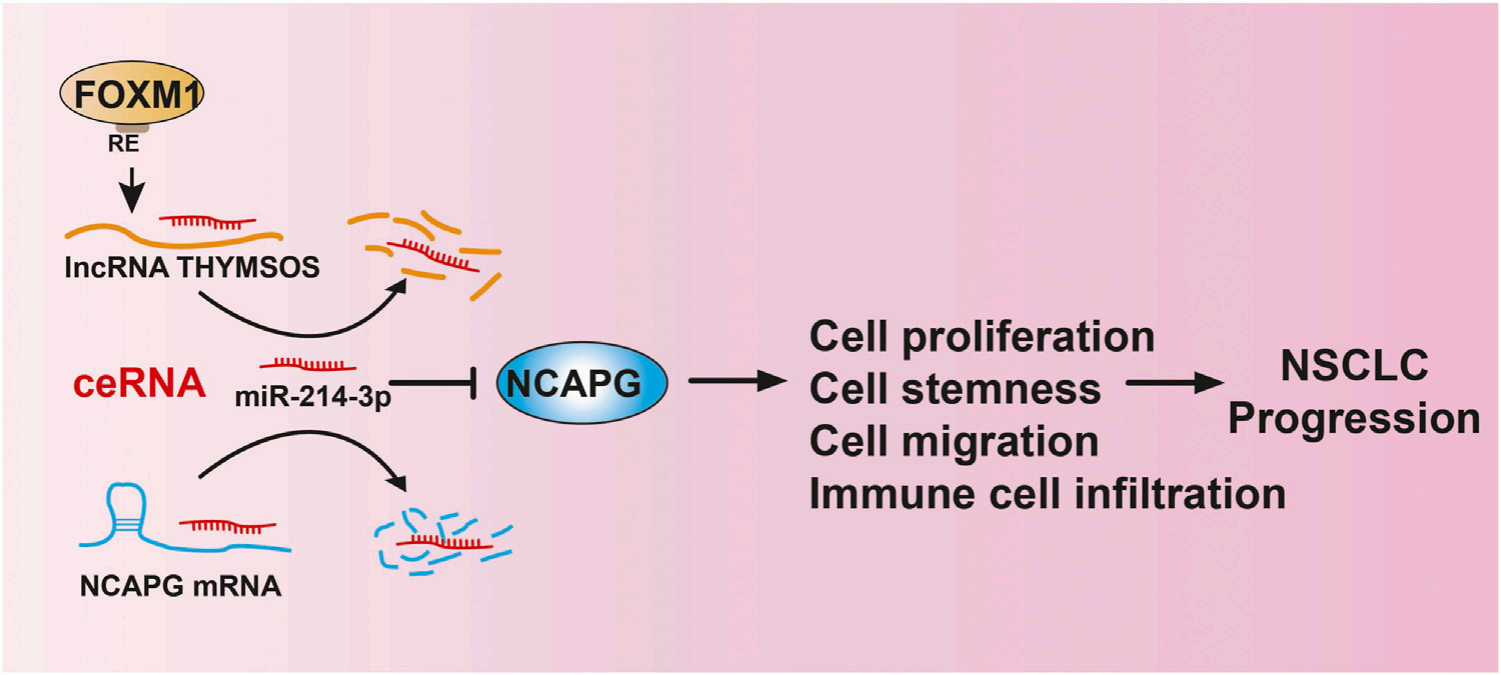
LncRNA-TYMSOS/miRNA-214-3p/NCAPG axis facilitated NSCLC progression.

We revealed that NCAPG was up-regulated in different cancers and positively correlated to poor prognosis in NSCLC. The upstream complex molecular regulation mechanism of NCAPG was first revealed by us, that is, FOXM1/TYMSOS/miRNA-214-3p (Figure 13). Furthermore, our work demonstrates that NCAPG was further implicated in the alteration of the tumor microenvironment. TYMSOS and NCAPG knockdown inhibited cell proliferation, cell migration, and cancer stem cell self-renewal in A549 cells. These findings suggest that NCAPG may play an oncogene role in the progression of lung cancer and represent an effective target of immunotherapy in the NSCLC.

To the best of our knowledge, this is the first study to explore the correlation between NCAPG and NSCLC. However, there are some limitations to our research. First, our study was based on expression data extracted from TCGA but may be more convincing if supported by a prospective clinical study. Furthermore, the biological functions of NCAPG need to be further explored in *in vivo* experiments. In the future, we will pay more attention to the function of NCAPG in tumor progression and tumor microenvironment regulation of NSCLC. Furthermore, we will perform more *in vivo* and *in vitro* experiments to explore the function and the potential molecular mechanisms of NCAPG in tumor progression and tumor microenvironment regulation of NSCLC.

## Conclusion

Our study found that NCAPG expression was increased in NSCLC, which was also associated with a poor prognosis. Furthermore, NCAPG might be involved in the progression of NSCLC by regulating the function of immune-infiltrating cells and immune response–related signaling pathways. Herein, we revealed the biological functions of NCAPG in NSCLC and offered a potential strategy for the diagnosis and treatment of NSCLC.

## Data Availability

The original contributions presented in the study are included in the article/Supplementary Material, further inquiries can be directed to the corresponding authors.

## Abbreviations

ACC, adrenocortical carcinoma; BLCA, bladder urothelial carcinoma; BRCA, breast invasive carcinoma; CESC, cervical squamous cell carcinoma and endocervical adenocarcinoma; COAD, colon adenocarcinoma; CSC, cancer stem cell; ESCA, esophageal carcinoma; GBM, glioblastoma multiforme; HNSC, head and neck squamous cell carcinoma; KICH, kidney chromophobe; KIRC, kidney renal clear cell carcinoma; KIRP, kidney renal papillary cell carcinoma; LAML, acute myeloid leukemia; LGG, brain lower grade glioma; LIHC, liver hepatocellular carcinoma; LUAD, lung adenocarcinoma; LUSC, lung squamous cell carcinoma; MESO, mesothelioma; MSI, microsatellite instability; OV, ovarian serous cystadenocarcinoma; PAAD, pancreatic adenocarcinoma; PCPG, pheochromocytoma and paraganglioma; PRAD, prostate adenocarcinoma; READ, colon adenocarcinoma; READ, rectum adenocarcinoma; STAD, stomach adenocarcinoma; TGCT, testicular germ cell tumors; THCA, thyroid carcinoma; TMB, tumor mutational burden; UCEC, uterine corpus endometrial carcinoma; UCS, uterine carcinosarcoma.
