# Supplementary material for: FOXM1/lncRNA TYMSOS/miR-214-3p–Mediated High Expression of NCAPG Correlates With Poor Prognosis and Cell Proliferation in Non–Small Cell Lung Carcinoma
Source: Front Mol Biosci. 2022 Feb 8;8:785767. doi: 10.3389/fmolb.2021.785767 (PMC8862726; doi:10.3389/fmolb.2021.785767)
Supplement: Supplementary file 1 [file DataSheet1.docx]

Supplementary Material

**
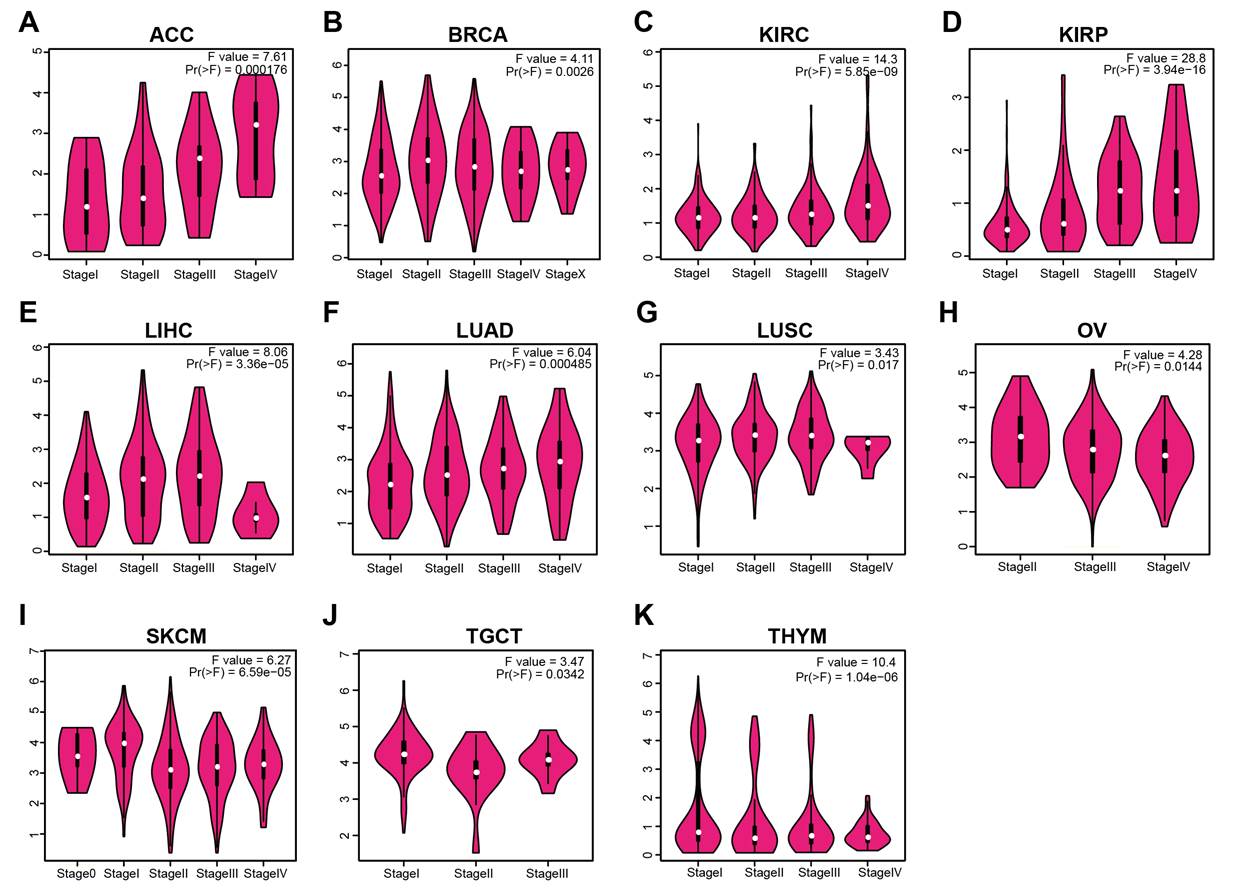
**

**Supplementary FIGURE 1 ⎜** **the pathological stage analysis for NCAPG in human cancers**

(A–K) The pathological stage of NCAPG in ACC (A), BRCA (B), KIRC(C), KIRP (D), LIHC(E), LUAD (F), LUSC(G), OV(H), SKCM(I), TGCT (J) and THCA (K) examined by GEPIA database.

**
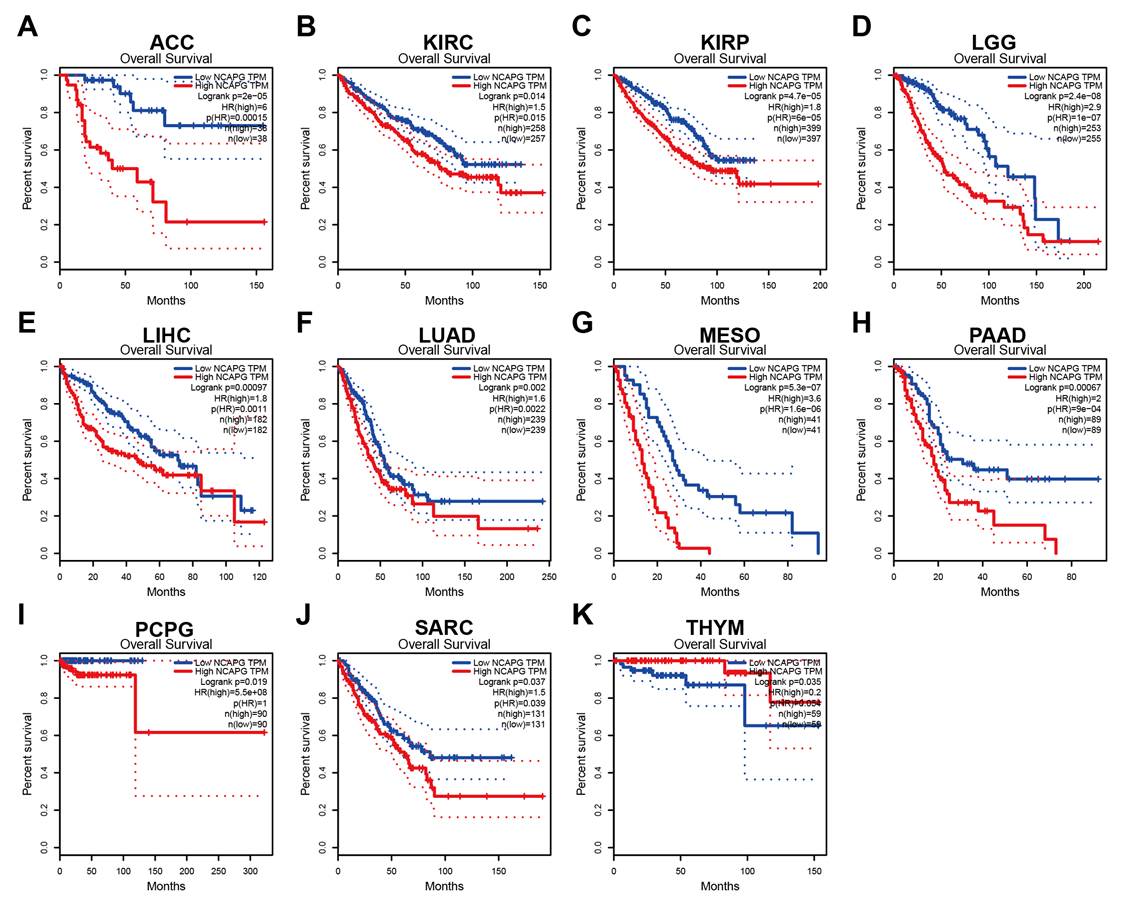
**

**Supplementary FIGURE 2 ⎜ the overall survival of NCAPG in diverse cancer.**

(A-K) The overall survival of NCAPG in ACC (A), KIRC (B), KIRP (C), LGG(D), LIHC (E), LUAD (F), MESO (G),PAAD (H),PCPG (I), SARC(J) and THYM(K) examined by GEPIA database.

**
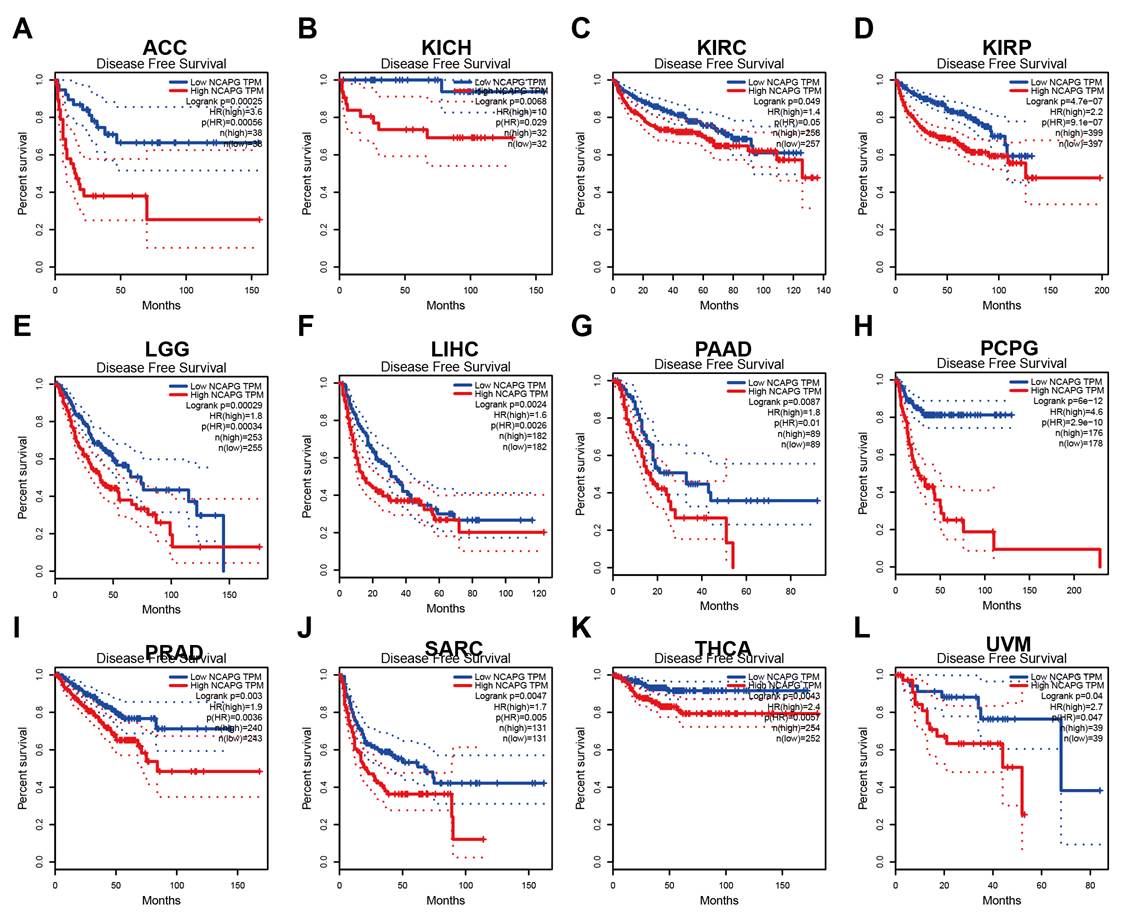
**

**Supplementary FIGURE 3 ⎜** **the Disease free survival of NCAPG in diverse cancer.**

(A-K) The disease free survival of NCAPG in ACC (A), KICH (B), KIRC (C), KIRP(D), LGG (E), LIHC (F), PAAD (G),PCPG (H),PRAD (I), SARC(J) , THCA(K) and THYM (L)examined by GEPIA database.


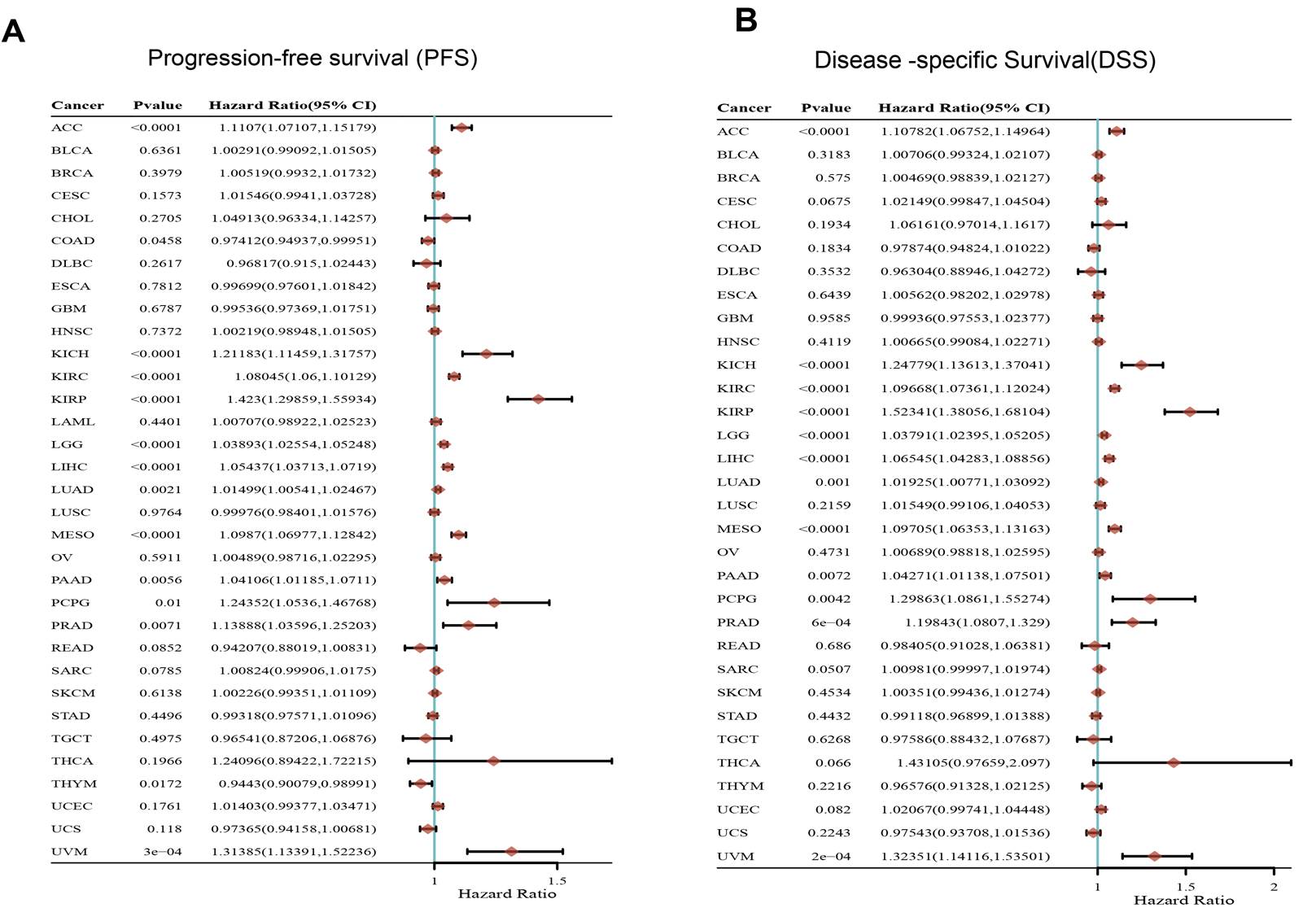


**Supplementary FIGURE 4 ⎜ Analysis the PFS and DSS of NCAPG in human cancer.**

(A-B) The PFS and DSS of NCAPG in diverse human cancers.


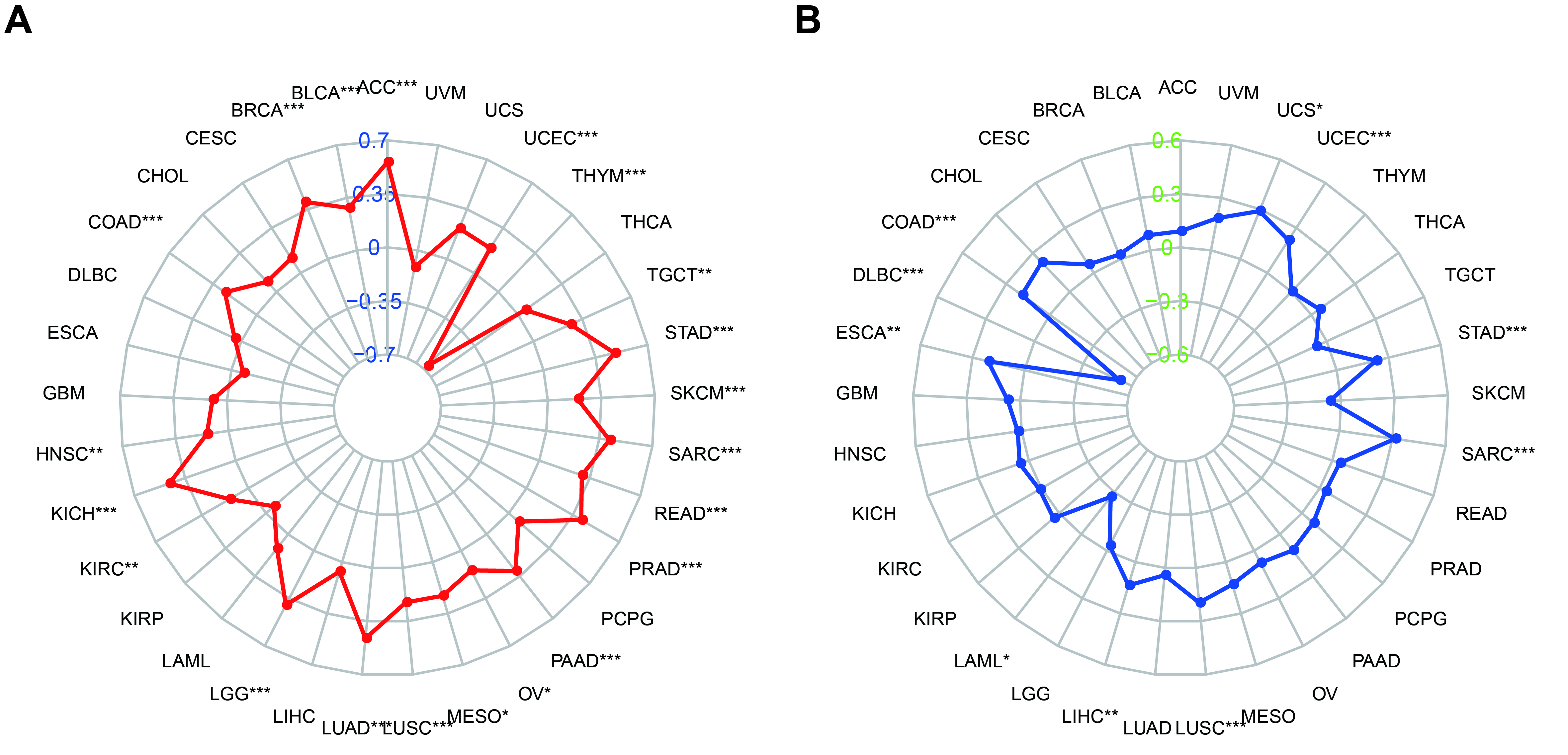


**Supplementary FIGURE 5 ⎜** The correlations between NCAPG expression and tumor mutation burden (TMB), and microsatellite instability (MSI) (A) The correlations between NCAPG expression and TMB in TCGA 33 types of cancers.(B) The correlation between NCAPG and MSI in TCGA 33 types of cancers.


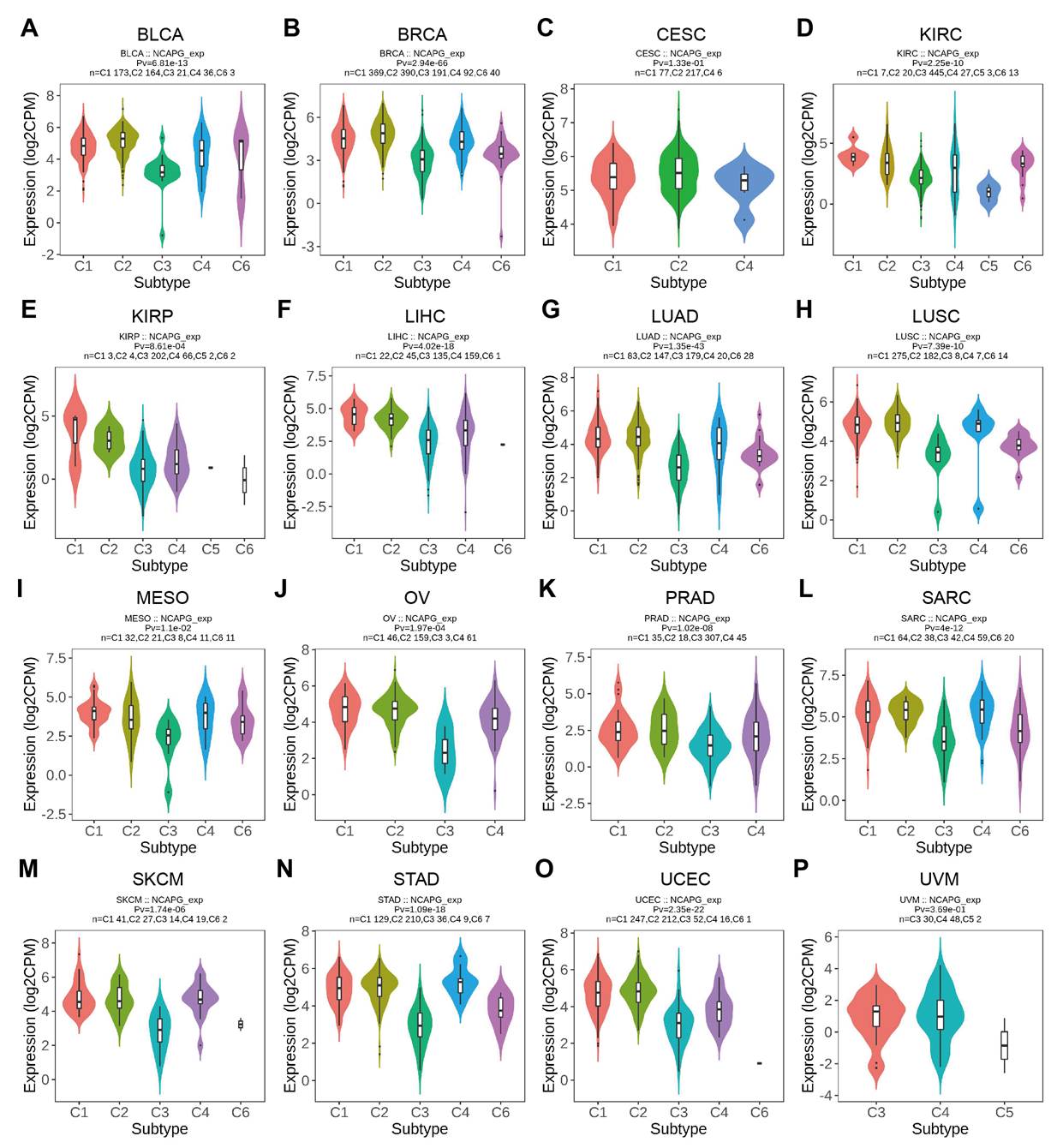


**Supplementary FIGURE 6 ⎜** The relationship between NCAPG expression and pan-cancer immune subtypes.

(A) in BLCA, (B) in BRCA, (C) in CESC, (D) in KIRC, (E) in KIRP, (F) in LIHC, (G) in LUAD, (H) in LUSC, (I) in MESO, (J) in OV, (K) in PRAD, (L) in SARC, (L) in SARC. (M) in SKCM. (N) in STAD. (L) in UCEC and (P) in UVM.


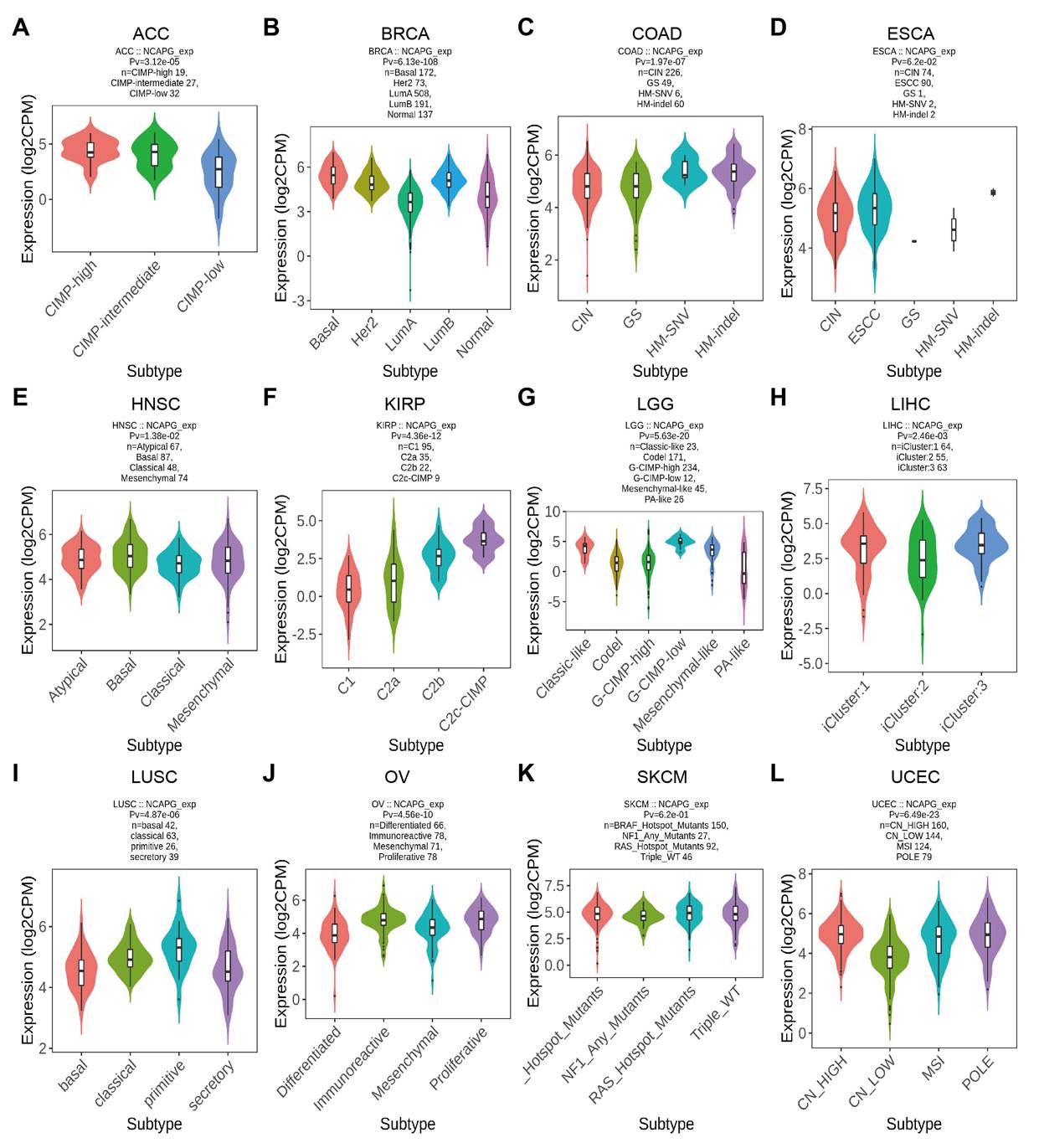


**Supplementary FIGURE 7 ⎜** The relationship between NCAPG expression and pan-cancer molecular subtypes. (A) in BRCA, (B) in BRCA, (C) in COAD, (D) in ESCA, (E) in HNSC, (F) in KIRP, (G) in LGG, (H) in LIHC, (I) in LUSC, (J) in OV, (K) in SKCM, (L) in UCEC.


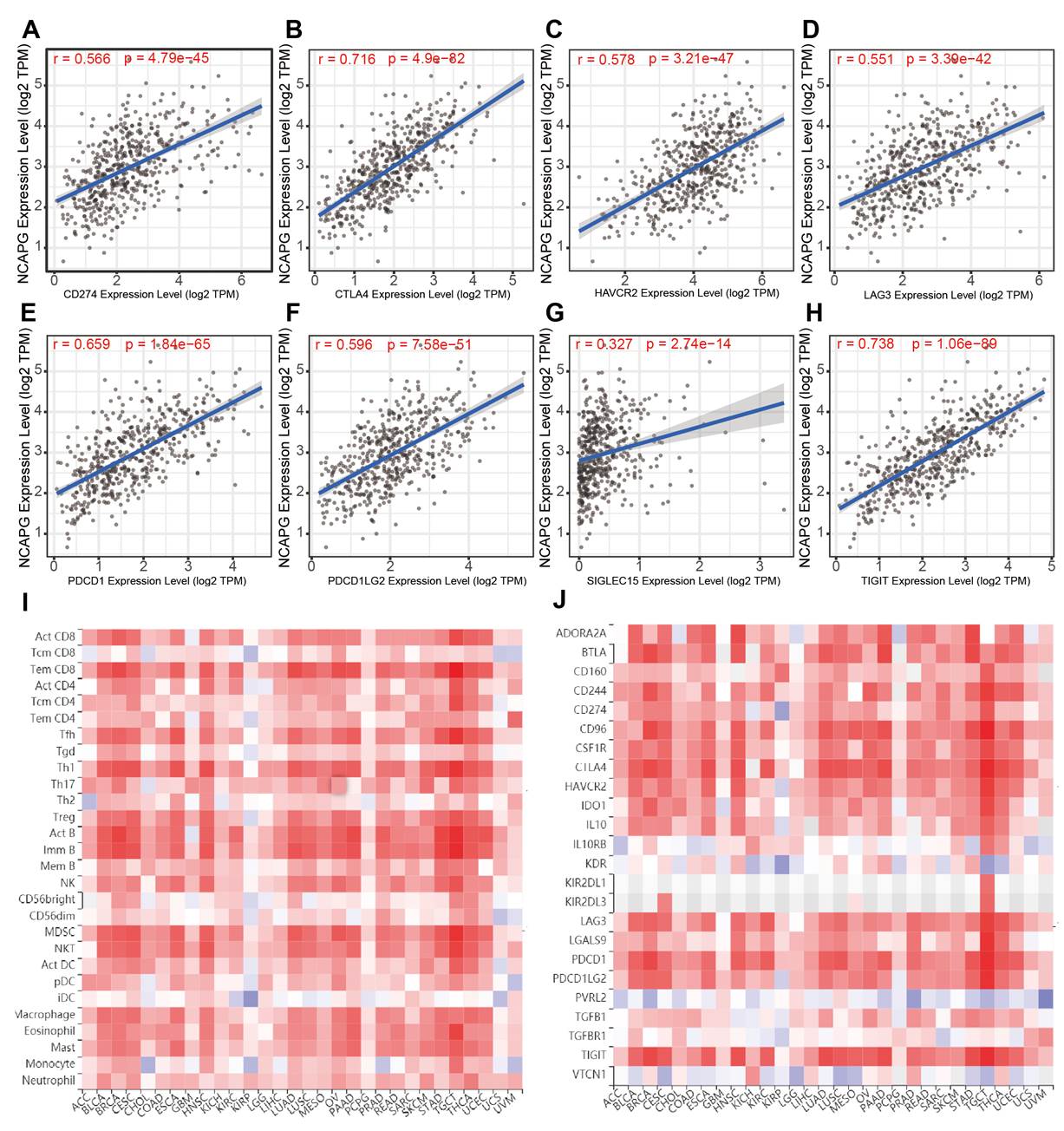


**Supplementary FIGURE 8 ⎜Correlation between NCAPG Expression and Various Immune checkpoints related gene**

(A-H)The correlations between NCAPG expression and different immune checkpoints related gene examined by the TIMER database. (I) The correlation between NCAPG expression and 28 TILs across human cancers examined by the TISIDB database. (J) The correlation between NCAPG expression and 24 immunoinhibitors across human cancers examined by the TISIDB database.
